# Supplementary material for: Novel regulators of heparan sulfate proteoglycans modulate cellular uptake of α-synuclein fibrils
Source: Commun Biol. 2025 Oct 6;8:1426. doi: 10.1038/s42003-025-08786-2 (PMC12501064; doi:10.1038/s42003-025-08786-2)
Supplement: Supplementary file 2 — Supplementary Information [file 42003_2025_8786_MOESM2_ESM.pdf]

<u>Title:</u> Novel regulators of heparan sulfate proteoglycans modulate cellular uptake of α-synuclein fibrils

Supplementary Figures

Authors:

Benoît Vanderperre [1,2,3,4,#*], Amitha Muraleedharan[1,2,3,*], Marie-France Dorion[5], Frédérique Larroquette[4], Esther Del Cid Pellitero[4], Nishani Rajakulendran[6], Carol X.-Q. Chen[5], Roxanne Larivière[4], Charlotte Michaud-Tardif[4], Thomas Goiran[4], Rony Chidiac[6], Damien Lipuma[1,2,3], Graham MacLeod[6], Rhalena Thomas [4,5], Zhangjie Wang[7], Wolfgang E. Reintsch[5], Wen Luo[5], Irina Shlaifer[5], Zhang Fuming[8], Ke Xia[8], Zachary Steinhart[6], Robert J. Linhardt[8,9], Jean-François Trempe[10], Jian Liu[7], Thomas M. Durcan[5], Stephane Angers[6], Edward A. Fon[4,#]

Affiliations:

[1]Département des Sciences biologiques, Université du Québec à Montréal, Montréal, QC, Canada.
[2]Centre d'Excellence de Recherche sur les Maladies Orphelines – Fondation Courtois (CERMO-FC), Montréal, QC, Canada.
[3]Network for Research on Protein Function, Engineering and Applications (PROTEO), Montréal, Quebec, Canada
[4]McGill Parkinson Program, Department of Neurology and Neurosurgery, Montreal Neurological Institute and Hospital, McGill University, Montréal, QC, Canada.
[5]The Neuro's Early Drug Discovery Unit (EDDU), Department of Neurology and Neurosurgery, Montreal Neurological Institute-Hospital, McGill University, Montréal, QC, Canada.
[6]Leslie Dan Faculty of Pharmacy, University of Toronto, Toronto, ON, Canada.
[7]Division of Chemical Biology & Medicinal Chemistry, Eshelman School of Pharmacy, University of North Carolina at Chapel Hill, Chapel Hill, NC, United States of America.
[8]Department of Chemistry and Chemical Biology, Rensselaer Polytechnic Institute, Troy, NY, Unites States of America.
[9]Deceased: Robert J. Linhardt.
[10]Department of Pharmacology & Therapeutics and Centre de Recherche en Biologie Structurale, McGill University, Montréal, QC, Canada.
[#]corresponding authors: vanderperre.benoit@uqam.ca; ted.fon@mcgill.ca
*These authors contributed equally

Keywords: alpha synuclein, CRISPR/Cas9, genome-wide screen, Parkinson's disease, heparan sulfate proteoglycans, preformed fibrils, protein aggregates

Supplementary Figure 1 – Recombinant α-syn-PFFs characterization

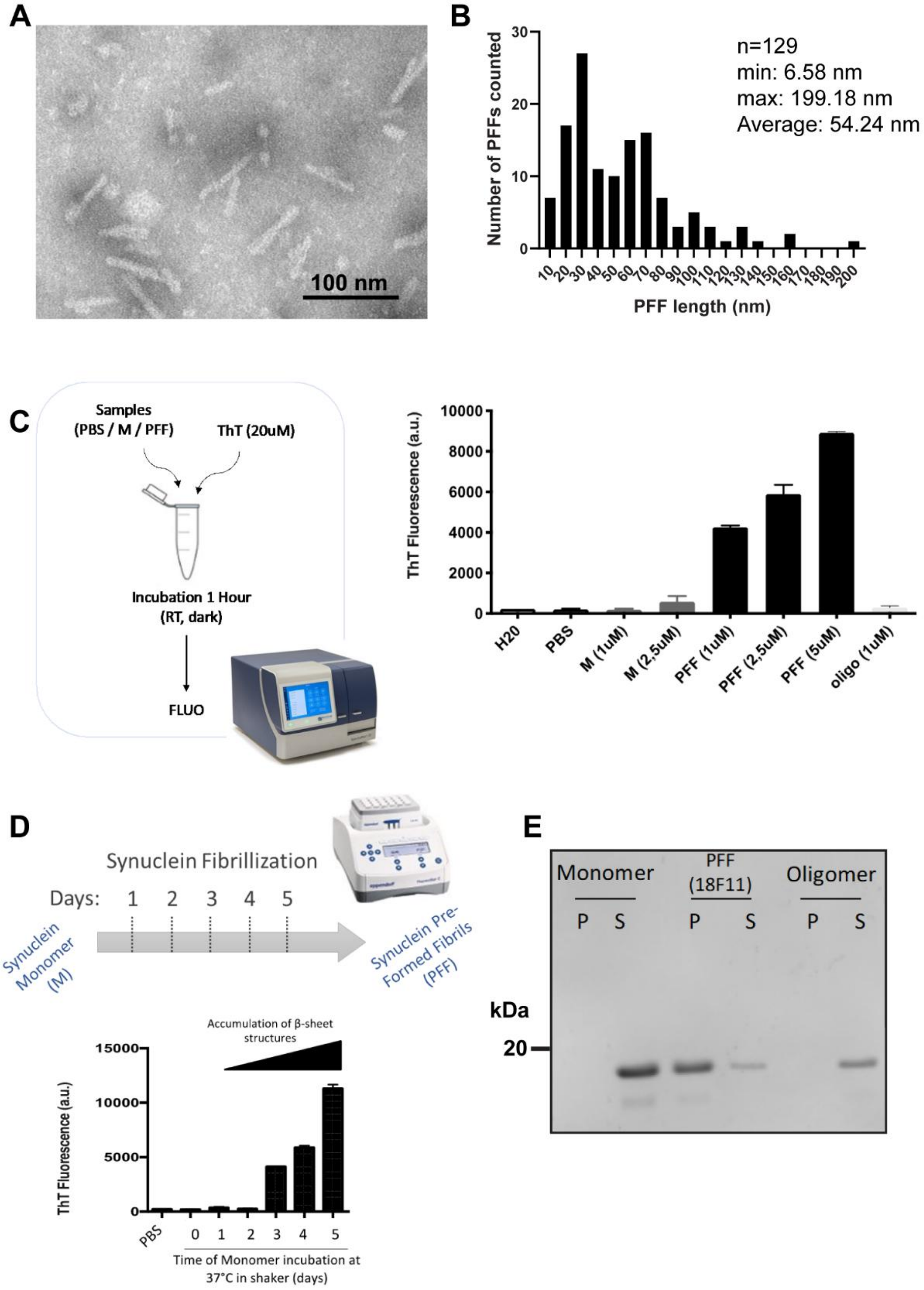

(A) Recombinant α-syn-PFFs purified from E. coli were stained with 2 % Uranyl Acetate and analyzed in a Tecnai 12 120 kV TEM microscope. **(B)** The length of PFFs was measured using ImageJ and the length distribution was obtained using MatLab. **(C)** ThT fluorescence assay showing formation of beta-sheet rich amyloid structures in the PFFs preparations, with ThT signal increasing in a concentration-dependent manner. Monomers and oligomers samples did not display significant ThT fluorescence. **(D)** Time-dependent increase in ThT fluorescence during the fibrillization process of PFFs generation. **(E)** Sedimentation assay by ultracentrifugation indicates that PFFs preparations used were mostly composed of insoluble material, while monomers and oligomers were entirely soluble.

Supplementary Figure 2 – Cell size is a confounding factor in FACS-based genome-wide CRISPR screening

The fact that most "inhibitors" of α-syn PFFs accumulation were related to cell growth/cell cycle processes **(Figure 1C)** pointed us toward a possible cell size-related bias in our screen. This was confirmed by several lines of evidence. FACS analysis of the cellular pool from the screen treated with fluorescent α-syn PFFs showed a significant correlation (R=0.764) between PFFs content and the FSC value, which measures cell size **(Supplementary Figure 2B)**. This indicates that any treatment that will modify cell size is likely to affect total PFFs content. In actively proliferating RPE-1 cells, this is likely to occur whenever cell cycle progression is affected. In our pooled genome-wide screen, this can occur upon disruption of cell cycle or cell growth-related genes. Accordingly, disruption of *TP53* and *CDKN1A* tumor suppressor genes is known to lead to increased cell proliferation due to defective G1/S checkpoint[1,2]. This would result in smaller cells, which would in turn have a lower PFFs content **(Figure 1B)**. Inversely, inactivation of oncogenes (*KIF11*, *MTBP*, *POLE2*, *TOP2A*,…) would result in slower cell-cycle progression (due to cell cycle blockade at the G2/M checkpoint for instance (*TOP2A*[3], *POLE2*[4])), and an overall increased cell size that would correlate with increased PFFs content, and thus enrichment of target sgRNAs in the "High PFF" population. Beyond the inactivation of specific cell cycle-related genes, it was previously shown that double-strand breaks (DSBs) induced by the CRISPR/Cas9 system itself lead to cell cycle arrest in a p53-dependent manner, independent of the target locus[5]. This likely explains the enrichment for non-targeting sgRNAs from the library (*LacZ*, *EGFP*, *Luciferase*, and Y chromosome-targeting in the XX chromosome-bearing RPE-1 cell line) in the "Low PFF" population, since such Cas9/sgRNA complexes are incompetent for DSB formation in the absence of the target DNA sequence **(Supplementary Figure 2C)**. The absence of the DSB-associated DNA damage response in these cells compared to all other cells from the pooled genome-edited population likely allows them to cycle faster relative to other cells, correlating with a reduced size and lower PFFs content. This was experimentally confirmed by comparing a cell line expressing *LacZ* sgRNA/Cas9 complexes to the pooled genome-edited population **(Supplementary Figure 2A)**. *LacZ* sgRNA-bearing cells had lower FSC value, and upon PFFs treatment, their median PFFs fluorescence value was well below the lower quartile of the pooled genome-edited population, in line with their enrichment in the "Low PFF" population upon FACS sorting based solely on PFFs fluorescence. The PFFs fluorescence/FSC ratio however shows that *LacZ* sgRNA-bearing cells have PFFs amount relative to cell size that is comparable with the pooled population. Together, this data demonstrates the importance of normalizing fluorescent signals to cell size in FACS-based genome-wide screens.

Supplementary Figure 2 (continued)

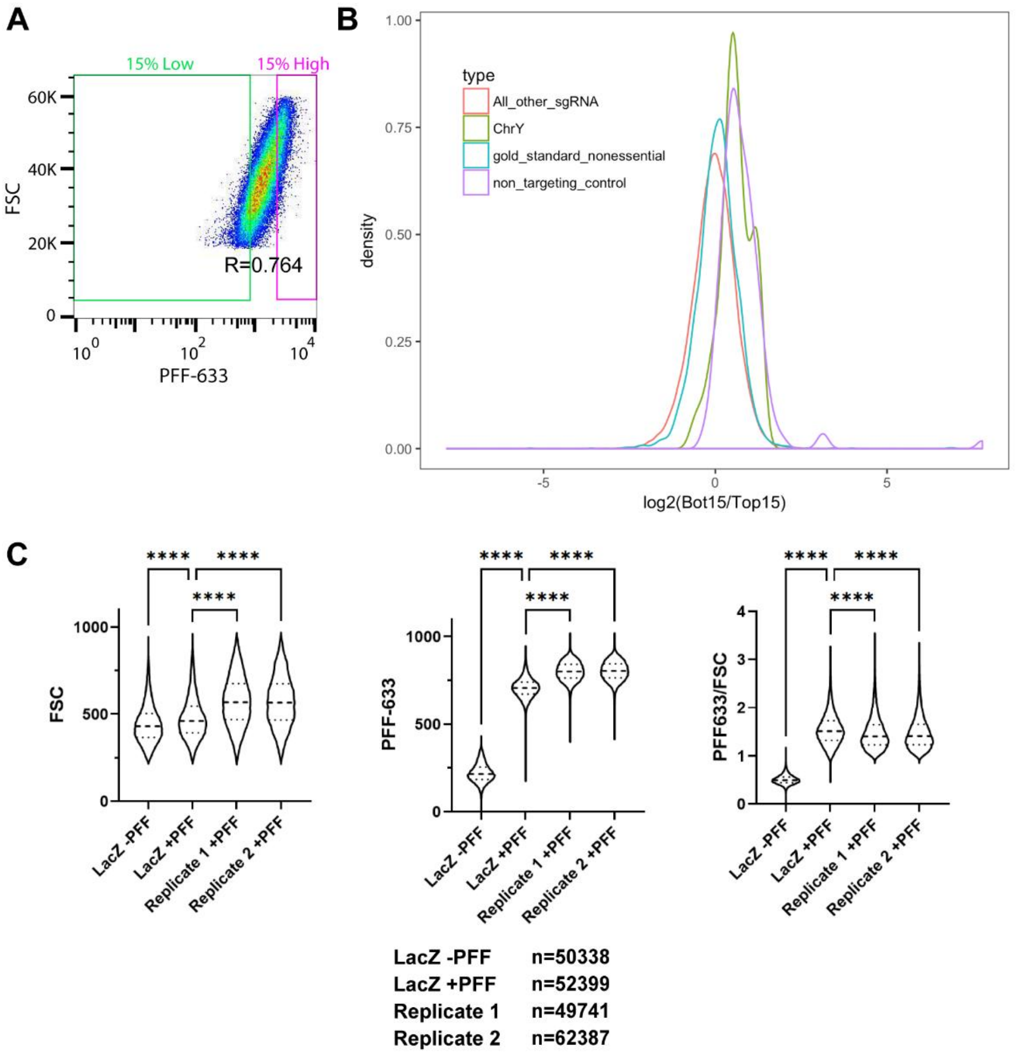

(A) Dot plot of FSC values as a function of PFFs-A633 fluorescence for cells transduced with the genome-wide CRISPR library. Linear regression was performed and the correlation coefficient (R) was calculated, showing a significant correlation between cell size and PFFs-A633 fluorescence. n=45455 cells from replicate 1 from the screen. **(B)** Densities of indicated sgRNA groups enrichment in the Low PFFs population versus the High PFFs population, showing that sgRNAs that don't result in double-strand break (non-targeting control and ChrY targeting sgRNAs in RPE-1 cells from female origin) are enriched in the Low PFFs population, most probably as a result of a lower cell size (see **(C)**). **(C)** Violin plots showing FSC (left), PFFs-A633 (middle), and PFFs-A633/FSC (right) values distribution for n>49500 cells per condition from samples analyzed by FACS at the end of the genome-wide CRISPR screen. A control population transduced with a non-targeting control sgRNA (*LacZ*) was untreated or treated with 15 nM PFFs-A633 at the same time as the two replicate populations from the actual genome-wide CRISPR library-transduced cells. The dashed lines and dotted lines indicate the median values and quartiles respectively. Statistical test: one-way ANOVA ; **** p<0.0001.

Supplementary Figure 3 – Hits selection, validation of screen validation methodology, and α-syn-PFFs uptake quantification in polyclonal KO cells for validated hits

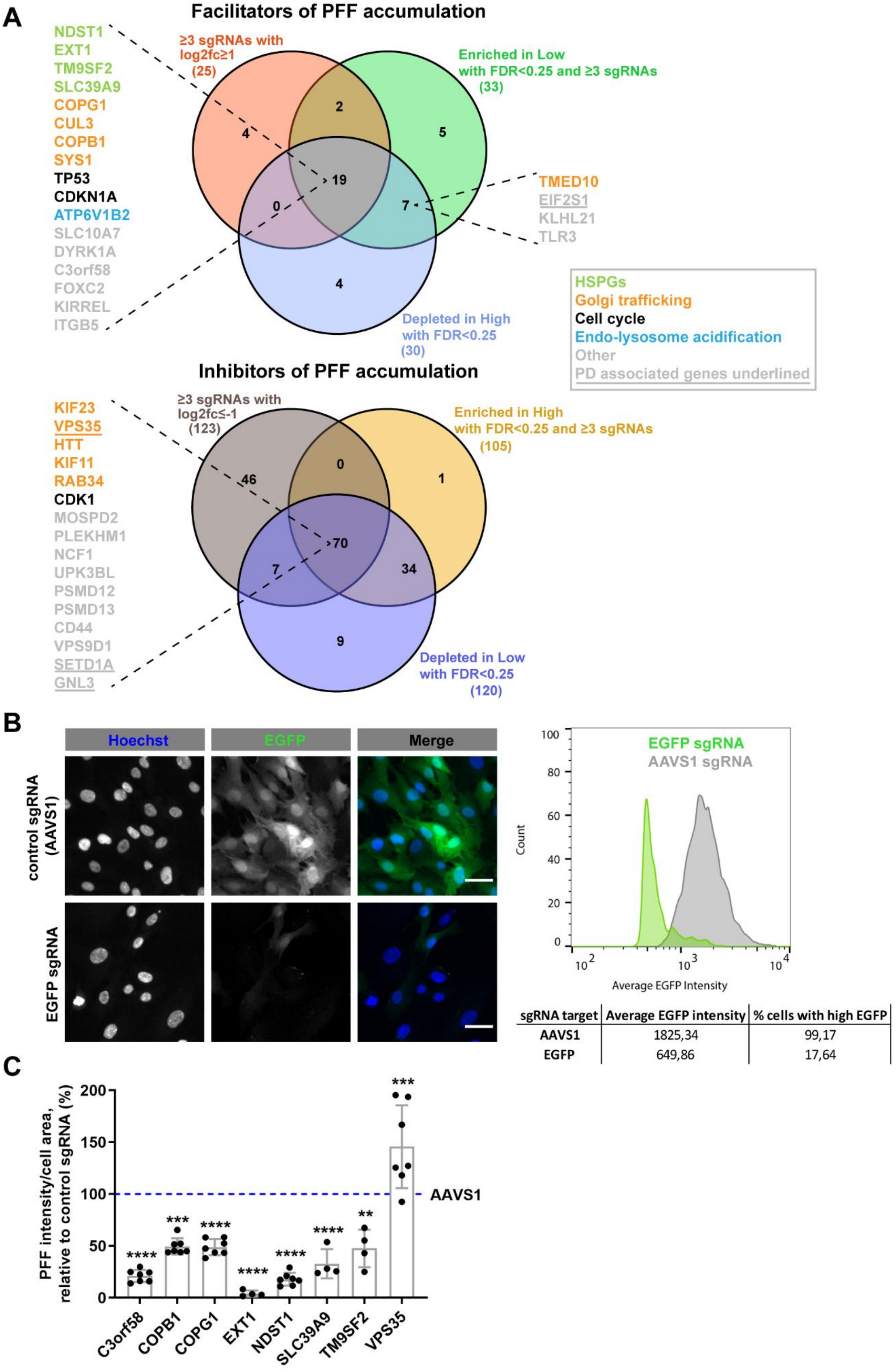

sgRNA target	Average EGFP intensity	% cells with high EGFP
AAVS1	1825,34	99,17
EGFP	649,86	17,64

Supplementary Figure 3 (continued)

(A) Based on MAGeCK sgRNA enrichment analysis (see Supplementary Tables S1, S2, and S3) between the low PFFs vs high PFFs populations, and the high PFFs vs low PFFs populations counter analysis, we selected hits to further validate that met the following criteria: 1) 3 or 4 sgRNAs with log2 fold-change ≥1 (for facilitators of PFFs uptake) or ≤-1 (for inhibitors of PFFs uptake); 2) enrichment in the low or high PFFs population with FDR <0.25 and 3 or 4 "good sgRNAs" ; 3) depletion in the high or low PFFs population with FDR <0.25 and 3 or 4 "good sgRNAs". Further filtering was done to remove most genes with ontologies related to cell growth or cell cycle (based on results from GOrilla analysis). Some hits were also cherry-picked with relaxed criteria, because of their location in genetic loci associated with PD (*EIF2S1*), or their good ranking despite some sgRNAs having magnitude of log2 fold-change not reaching the ≥1 cut-off (*TMED10*, *KLHL21*, *TLR3*) **(B)** RPE-1 cells stably expressing Cas9 and EGFP were transfected with a control AAVS1-targeting or EGFP-targeting sgRNA for 3 days before fixation, nuclei staining with Hoechst 33342 and analysis using a CX7 high-content microscope. The average EGFP intensity per cell and % cells with high EGFP intensity were quantified (table on the right). The results of the cellular analysis were exported as FCS files, and the FlowJo software was used for graphic representation of both cell populations (*AAVS1* sgRNA-treated in grey, *EGFP* sgRNA-treated in green). Scale bars: 30 µm. **(C)** The results of PFFs intensity/cell area from Figure 2B (TKOv3 sgRNA) of the high-content microscopy-based validation are shown as a histogram for the indicated genes. n=4-7 independent experiments. Statistical test: one-way ANOVA; ** p<0.01, *** p<0.001, **** p<0.0001.

Supplementary Figure 4 – Validation for cargo specificity

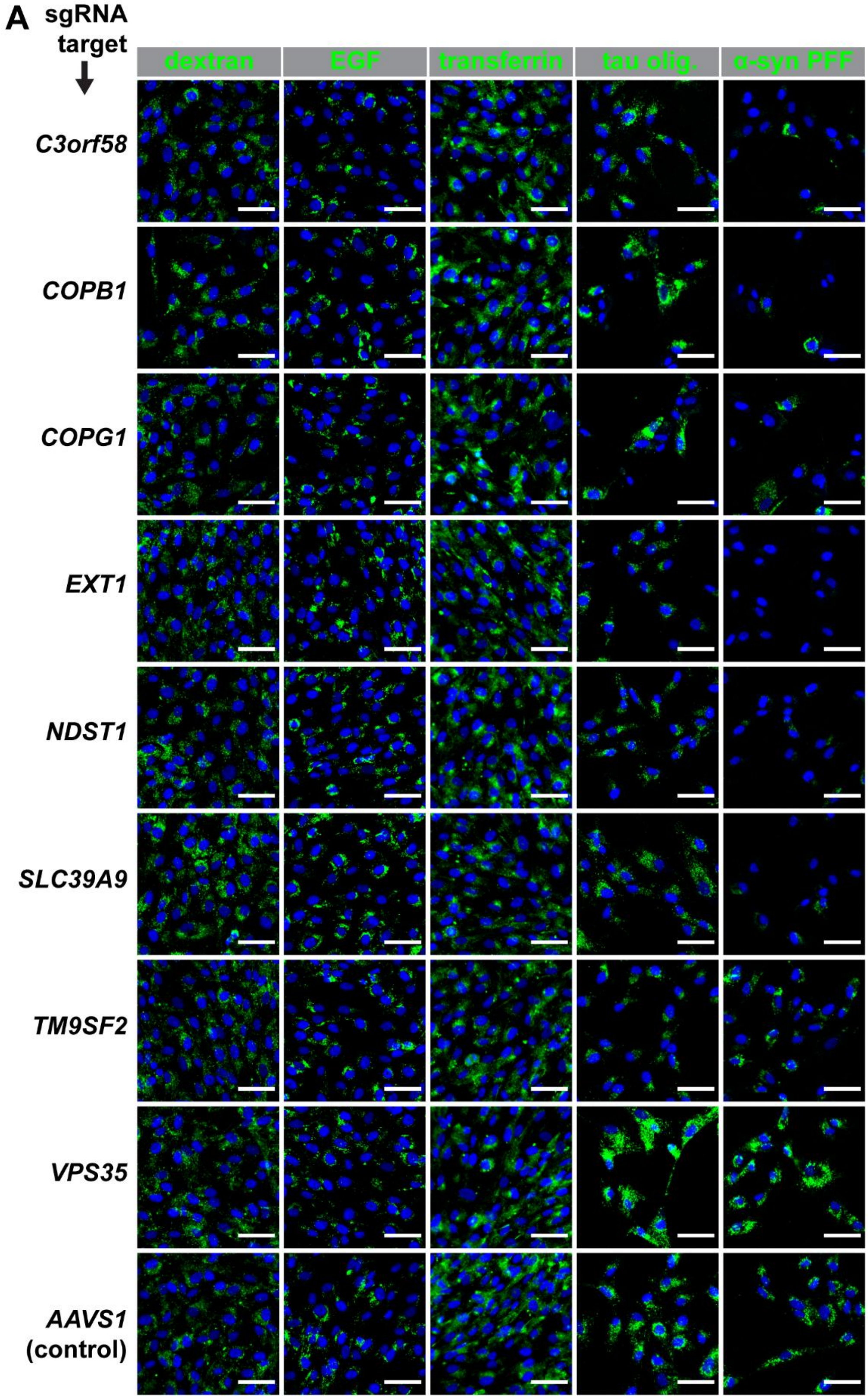

Supplementary Figure 4 (continued)

(A) RPE-1 cells stably expressing Cas9 nuclease were transfected in 96 wells plates with individual synthetic sgRNAs, and after 3 days, were subjected to 24 h uptake of the indicated cargoes (green): dextran-Oregon Green 488, EGF-A488, transferrin-A488, Tau oligomers-Cy3, and α-syn PFFs-A633 as positive controls of sgRNAs' effects. Cells were then fixed, and nuclei were stained with Hoechst 33342 (blue). Imaging was done using a CX7 high-content microscope. Quantifications are shown for each cargo in **Figure 3**. Scale bars: 30 µm.

Supplementary Figure 5 – sequences of monoclonal CRISPR-invalidated lines used

A

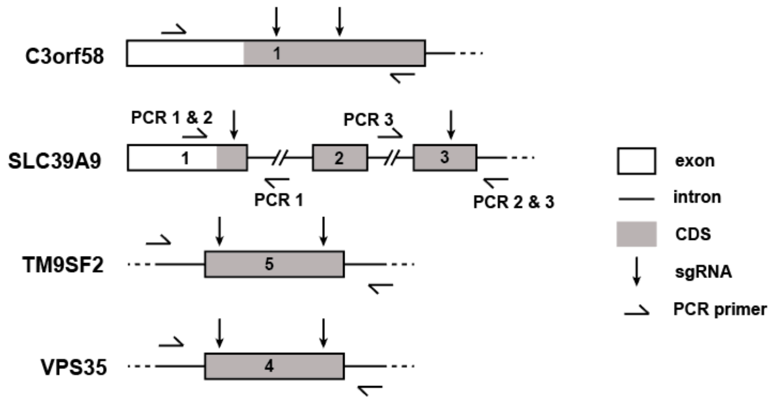

B *C3orf58*$^{-/-}$ clone 3

```
allele 1        MWRLVPPKLGRLSRSLKLAALGSLLVLMVLHSPSLLASWQRNELTPSGAQAPRL-AARAG    59
wt              MWRLVPPKLGRLSRSLKLAALGSLLVLMVLHSPSLLASWQRNELTDRRFLQLNKCPACFG    60
allele 2        MWRLVPPKLGRLSRSLKLAALGSLLVLMVLHSPSLLASWQRNELTRPALPAAQ*VPGVLR    59
                *********************************************       .   .

allele 1        AARPEHLQAGHRPA----PLRPAAGHAPDR--------VRAPQRRRASAHARGGGGLV--    105
wt              TSWCRRFLNGQVVFEAWGRLRLLDFLNVKNVYFAQYGEPREGGRRRVVLKRLGSQRELAQ    120
allele 2        ----------------------------------------------------------HE    61

allele 1        -----------GPGALPLAAPSRPPGAPLRGDQGLGQLPASQPQGLGAHAAAADPGLQPR    154
wt              LDQSICKRATGRPRCDLLQAMPRTEFARLNGDVRLL--TPEAVEGWSDLVHCPSQRLLD-    177
allele 2        LVPPLPQRAGGRPRCDLLQAMPRTEFARLNGDVRLL--TPEAVEGWSDLVHCPSQRLLD-    118
                    * .  * *  *   * *.**  *     .  :* .  . . .  *

allele 1        AAGATEFSV**RLAICKVSWSLWKNGGCKLCW-RR---TVE-LL*CAMGKTS*PRLAING    205
wt              -----------RL---VRRYAETKDSGSFLLRNLKDSERMQLLLTLAFNPEPLVLQSFPS    223
allele 2        -----------RL---VRRYAETKDSGSFLLRNLKDSERMQLLLTLAFNPEPLVLQSFPS    164
                   **      ::  *:.*. *    :    :: **  *:.       :: .

allele 1        NSRTAYKQ*L*ICTLPPGRQL*---QFCSWS*RWEGNHCGC*KCFGC*QKIN*TK*T*KL    253
wt              DEGWPFAKYLGACGRMVAVNYVGEELWSYFNAPWEKRVDLAWQLMEIAEQLTNNDFEFAL    283
allele 2        DEGWPFAKYLGACGRMVAVNYVGEELWSYFNAPWEKRVDLAWQLMEIAEQLTNNDFEFAL    224
                :.   : : *  *    . :      :. :.  ** .   . : :   :::. ..    *

allele 1        GC--MV*KQV**L**GGLLIIFKRNSLCSCHCGPQLLCCLSEPLIQTCHLAWHFWRTPS*    305
wt              YLLDVSFDNFAVGPRDGKVIIVDAEN---------VLVADKRLIRQNKPENWDVWYESKF    334
allele 2        YLLDVSFDNFAVGPRDGKVIIVDAEN---------VLVADKRLIRQNKPENWDVWYESKF    275
                    :  .:.     .* :**.. :.         :* . .. : *.    *..*   .

allele 1        ST--K*NCQRWPARGL---------AG*VCQP---KEALWQIPGCKRTA*IPSTIK*QRE    347
wt              DDCDKEACLSFSKEILCARATVDHNYYAVCQNLLSRHATWRGTSGGLLHDPPSEIAKDGR    394
allele 2        DDCDKEACLSFSKEILCARATVDHNYYAVCQNLLSRHATWRGTSGGLLHDPPSEIAKDGR    335
                .   *  *  :  . *            ***    :.* *:  .       ** *  : .

allele 1        V-----------------------------------   348
wt              LEALLDECANPKKRYGRFQAAKELREYLAQLSNNVR*   430
allele 2        LEALLDECANPKKRYGRFQAAKELREYLAQLSNNVR*   371
                :
```

Supplementary Figure 5 (continued)

C *SLC39A9*$^{-/-}$ clone 10

```
allele 1      MDDFISISLLSLAMLVGCYVAGII-LAVNFSEERLKLVTVLGAGLLCGTALAVIVPEGVH    59
wt            MDDFISISLLSLAMLVGCYVAGIIPLAVNFSEERLKLVTVLGAGLLCGTALAVIVPEGVH    60
allele 2      MDDFISISLLSLAMLVGCYVAGII------------------------------------    24
              ***********************

allele 1      ALYEDILEGKHHQASETHNVIASDKAAEKSVVHEHEHSHDHTQLHAYIGVSCCWWTRLVT    119
wt            ALYEDILEGKHHQASETHNVIASDKAAEKSVVHEHEHSHDHTQLHAYIGVSLVLGFVFM-    119
allele 2      -----------------------------------------------------LLGFVFM-    31
                                                                     ::

allele 1      PMCILLTIQKQQGLA--IPKSPPRWVWL------SMLQLMVLLWEQQHLLHRPV-----S    166
wt            -----LLVD-QIGNSHVHSTDDPEAARSSNSKITTTLGLVVHAAADGVALGAAASTSQTS    173
allele 2      -----LLVD-QIGNSHVHSTDDPEAARSSNSKITTTLGLVVHAAADGVALGAAASTSQTS    85
                   * :: * * :    .. *. .        : * *:*    :   *   .     *

allele 1      S*LCLWQSCYIRHQLLLDWFPS*CMLA*SGIESESTCWSLHWQHQLCPW*HT---*D*VR    217
wt            VQLIVFVAIML-HKA-PAAFGLVSFLMHAGLERNRIRKHLLVFALAAPVMSMVTYLGLSK    231
allele 2      VQLIVFVAIML-HKA-PAAFGLVSFLMHAGLERNRIRKHLLVFALAAPVMSMVTYLGLSK    143
                * :: :  : *:     *   .:*  :*:* :     *      .*        .  :

allele 1      AVKKPFQR*TPREWPCFSLPGHFFMLPQYMSSLRWAE*GTATSPMPREGEASAAWKWQPW    275
wt            SSKEALSEVNATGVAMLFSAGTFLYVATVHVLPEVGGIGHSHKPDATGGRGLSRL-----    286
allele 2      SSKEALSEVNATGVAMLFSAGTFLYVATVHVLPEVGGIGHSHKPDATGGRGLSRL-----    198
              : *: :.. .      :   * *: :       . .  * : .*    *.. :

allele 1      FWVASSLSSCQ*DTSI-------  290
wt            -EVAALVLGCLIPLILSVGHQH*  307
allele 2      -EVAALVLGCLIPLILSVGHQH*  219
                **: : .*     :
```

D *TM9SF2*$^{-/-}$ clone 11

```
WT          MSARLPVLSPPRWPRLLLLSLLLLGAVPGPRRSGAFYLPGLAPVNFCDEEKKSDECKAEI        60
alleles 1+2 MSARLPVLSPPRWPRLLLLSLLLLGAVPGPRRSGAFYLPGLAPVNFCDEEKKSDECKAEI        60
            ************************************************************

WT          ELFVNRLDSVESVLPYEYTAFDFCQASEGKRPSENLGQVLFGERIEPSPYKFTFNKKETC        120
alleles 1+2 ELFVNRLDSVESVLPYEYTAFDFCQASEGKRPSENLGQVLFGERIEPSPYKFTFNKKETC        120
            ************************************************************

WT          KLVCTKTYHTEKAEDKQKLEFLKKSMLLNYQHHWIVDNMPVTWCYDVEDGQRFCNPGFPI        180
alleles 1+2 KLVCTKTYHTEKAEDKQKLEFLKKSMLLNYQHHWIVDNMPVLLVQISMKEIHFTSSTMLT        180
            *****************************************       .  :* .  :

WT          GCYITDKGHAKDACVISSDFHERDTFYIFNHVDIKIYYHVVETGSMGARLVAAKLEPKSF        240
alleles 1+2 SKYTI--------MLLKLGPWEQD*WL-------------------------LNLNRKAS        206
            . *           ::. .  *:* :                           :*: *:
```

Only the first 240 amino acids are shown.

E *VPS35*$^{+/-}$ clone 21

```
allele_1      MPTTQQSPQDEQEKLLDEAIQAVKVQSFQMKRCLDKNKLMDALKHASNMLGELRTSMLSP     60
WT            MPTTQQSPQDEQEKLLDEAIQAVKVQSFQMKRCLDKNKLMDALKHASNMLGELRTSMLSP     60
allele_2      MPTTQQSPQDEQEKLLDEAIQAVKVQSFQMKRCLDKNKLMDALKHASNMLGELRTSMLSP     60
              ************************************************************

allele_1      KSYYELYMAISDELHYLEVYLTDEFAKGRKVADLYELVQYAGNIIPRLYLLITVGVVYVK     120
WT            KSYYELYMAISDELHYLEVYLTDEFAKGRKVADLYELVQYAGNIIPRLYLLITVGVVYVK     120
allele_2      KSYYELYMAISDELHSVVCNIP*GVC-------FFEITFFS---VPEISYLM-------K     102
              *************** :   :   ..       ::*:. ::   :*.:  *:       *
```

Only the first 120 amino acids are shown. Allele 1 was identical to WT.

(A) Schematic representation of knock-out (KO) and PCR-screening strategies for the indicated genes in human cells. For all genes, allelic sequences from the indicated CRISPR-edited RPE-1 cell clones were PCR-amplified using Q5 polymerase and the indicated oligonucleotides. For *C3orf58* **(B)** and *SLC39A9* **(C)**, all PCR products were cloned using a PCR cloning kit, and 10 plasmids from individual bacterial clones were sequenced. For *TM9SF2* **(D)** and *VPS35* **(E)**, the PCR products were directly subjected to Sanger sequencing, and the resulting trace files were analyzed using ICE analysis (Synthego) against the WT sequences. The identified allelic sequences are shown and aligned to the reference WT sequence.

Supplementary Figure 6 – Heparan sulfate staining in monoclonal CRISPR -/- and/or rescue cells for *C3orf58*, *SLC39A9*, *TM9SF2,* and *VPS35* & PFFs uptake in monoclonal *TM9SF2* -/- and *VPS35* +/- cells

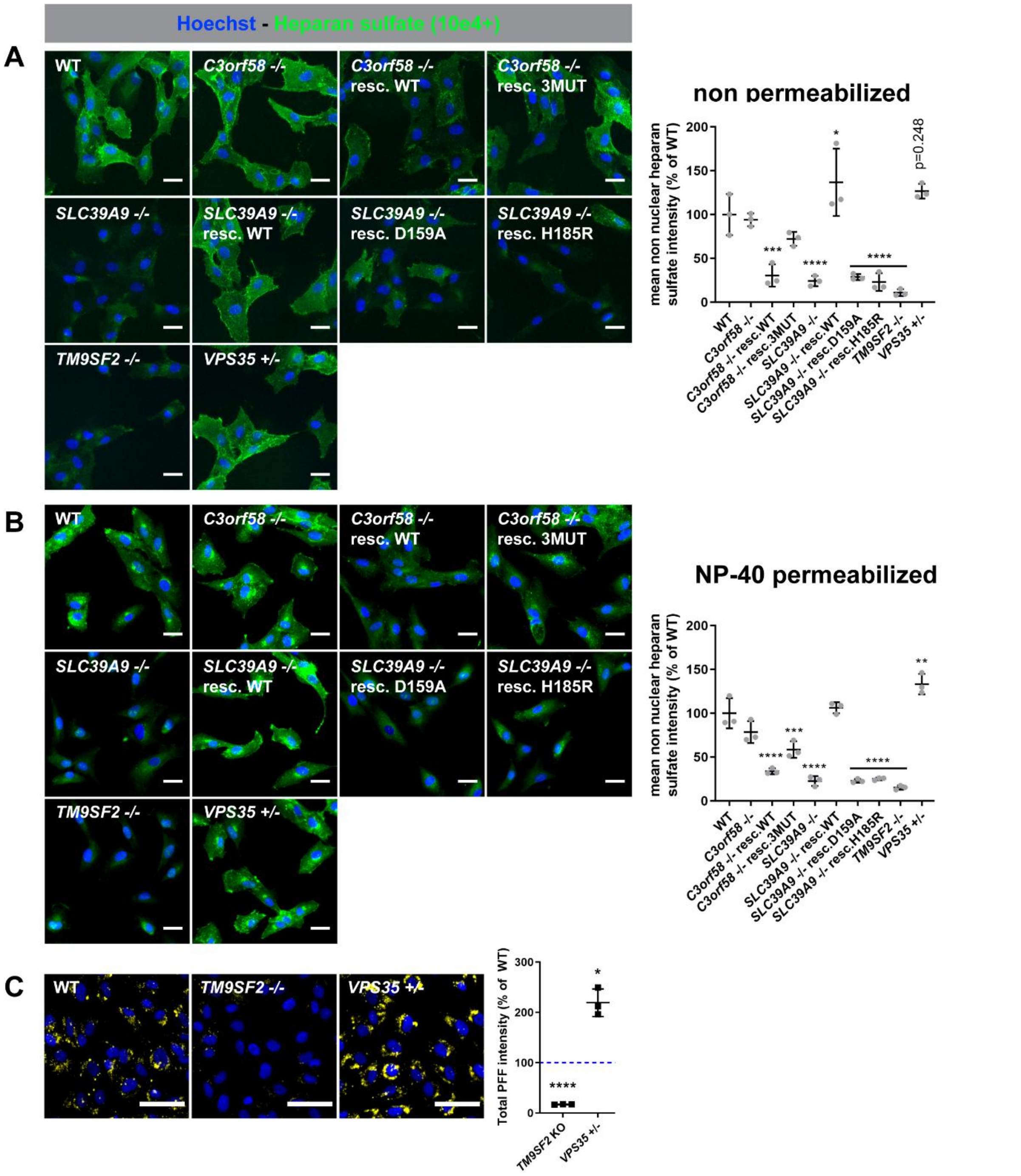

(A, B) RPE-1 cells of the indicated genotypes (± rescue constructs) were fixed, then processed for immunostaining against Heparan sulfate (antibody clone 10e4, green) either in non-permeabilized conditions **(A)** or following permeabilization with 0.05% NP-40 **(B)** to label surface or total heparan sulfate respectively. Nuclei were stained with Hoechst 33342 (blue). Scale bars: 30 µm. Cells were then imaged with a CX7 high-content microscope and analyzed with the HCS Studio Cell Analyzer software. Nuclei in the Hoechst channel were used for object identification, and the mean of the total non-nuclear heparan sulfate signal was quantified for each condition. Data in graphs are shown

as mean ± SD, n=3. **(C)** 24h uptake of 60 nM PFFs-A633 (yellow) in *TM9SF2* -/- or *VPS35* +/- RPE-1 cells, either in the absence or presence of stable expression of the indicated C-terminally HA-tagged rescue constructs. Nuclei were stained with Hoechst (blue), and the mean PFFs-A633 fluorescence intensity was quantified by high-content microscopy and normalized to WT RPE-1 cells (see graph, right panel). n=3 independent experiments. Statistical test: one-way ANOVA; * $p<0.05$, **** $p<0.0001$. Scale bars: 60 µm.

Supplementary Figure 7 – Localization of SLC39A9 and C3orf58 in RPE-1 cells

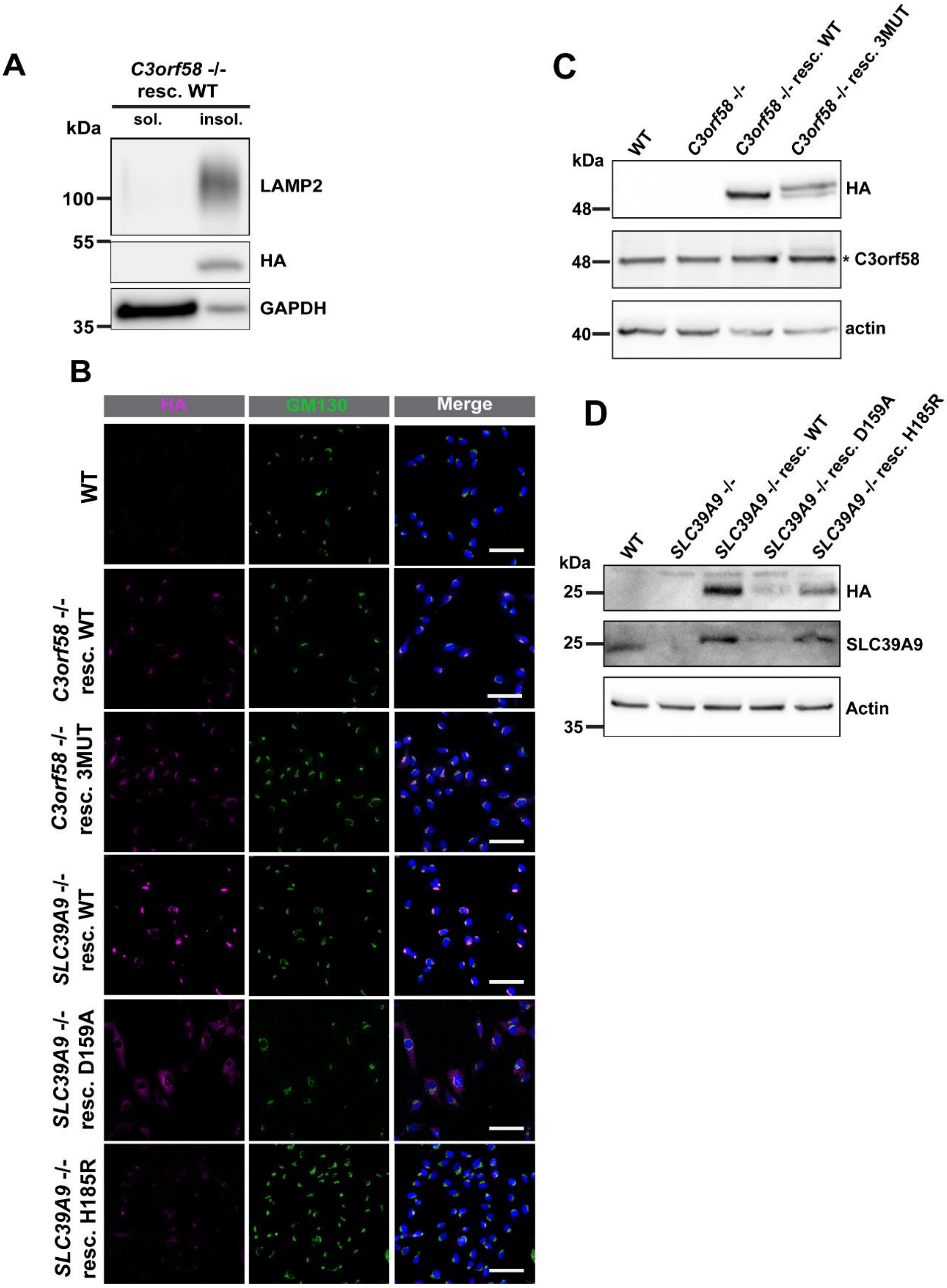

(A) Soluble and membrane-associated proteins (soluble fraction = sol.) from *C3orf58 -/-* RPE-1 cells rescued with WT C3orf58-HA were separated from integral membrane proteins (insoluble fraction = insol.) by 0.1 M sodium carbonate (pH 11.5) extraction followed by ultracentrifugation at 100,000 g. Both fractions were analyzed by Western blot against GAPDH (soluble protein), LAMP2 (transmembrane protein), and HA showing that C3orf58 is an integral membrane protein. **(B)** The *SLC39A9 -/-* or *C3orf58 -/-* were rescued using lentiviral transduction to drive puromycin-selectable constitutive expression of C-terminally HA-tagged constructs encoding WT SLC39A9, D159A or H185R

(Zn^{2+} transport-deficient mutants) SLC39A9, WT C3orf58 or triple mutant (putative kinase-dead, 3MUT) C3orf58 as indicated. These cells as well as WT RPE-1 cells were analyzed by immunofluorescence using anti-HA (magenta) and anti-GM130 (Golgi marker, green) antibodies. Nuclei were stained with Hoechst 33342 (blue), and images were acquired using a Nikon A1 laser scanning confocal microscope at 40x. Scale bars: 20 µm. **(C)** Western blot showing expression levels of HA-tagged C3orf58 WT and 3MUT in rescued monoclonal RPE-1 cells, while endogenous C3orf58 could not be detected using commercial antibodies (here, cat. 27145-1-AP, Proteintech; a non-specific band around the expected molecular weight was observed (asterisk)). **(D)** Western blot showing expression levels of HA-tagged SLC39A9 WT, D159A and H185R mutant constructs in rescued monoclonal RPE-1 cells. Endogenous expression is also shown in WT cells, which is lost in KO cells.

Supplementary Figure 8 – SLC39A9 modulates Zinc homeostasis in the Golgi

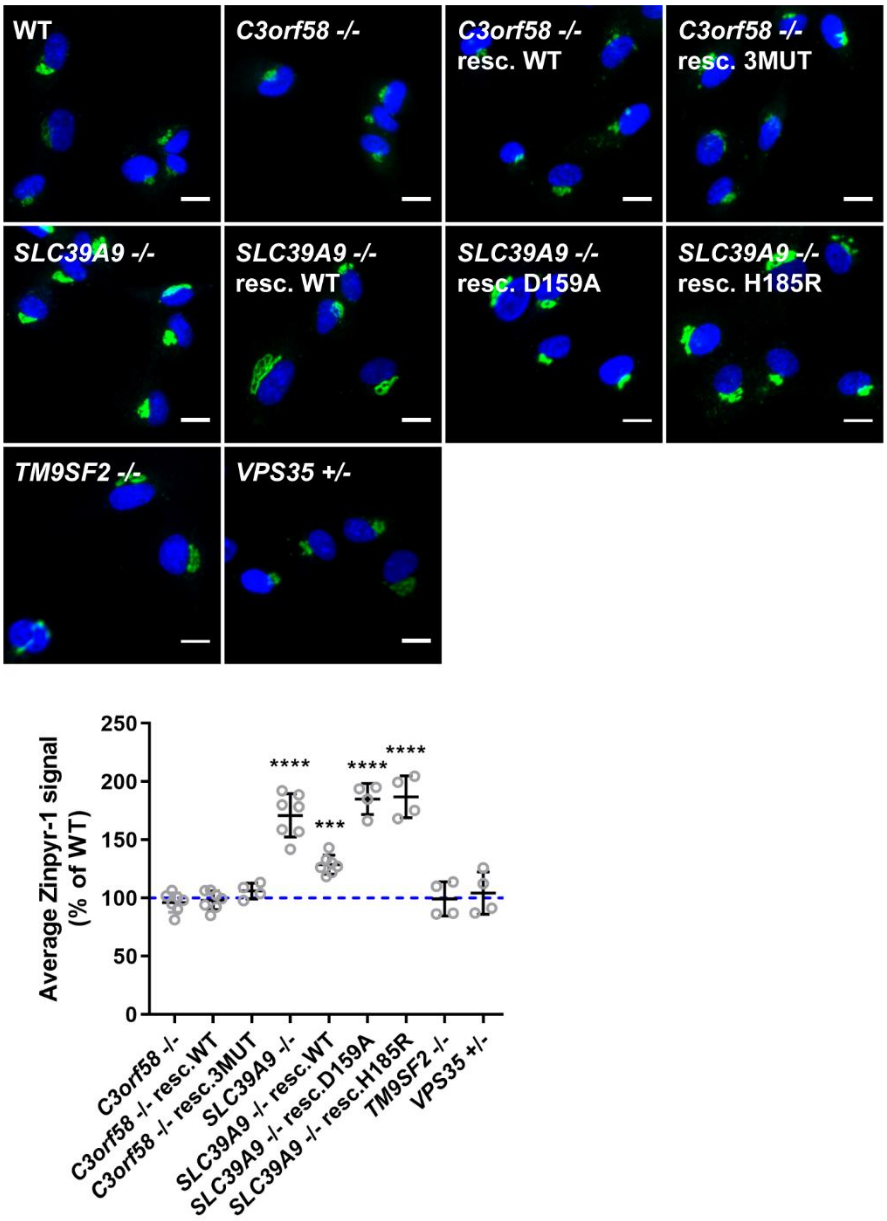

RPE-1 cells of the indicated genotypes were seeded in 96-well plates for imaging. One day later, cells were incubated at 37°C for 30 min with complete DMEM containing 5 µM Zinpyr-1 (green), Hoechst 33342 (blue), and 1 mM EDTA to chelate extracellular Zn^{2+}. After several washes in Live Cell Imaging Solution containing 1 mM EDTA, live cells were imaged with a CX7 high-content microscope, and the mean average intensity per cell was quantified with the HCS Studio Cell Analysis software (n=3, >300 cells per experiment per condition). Statistical test: one-way ANOVA, *** p<0.001, **** p<0.0001. Scale bars: 30 µm.

Supplementary Figure 9 – Tau oligomers uptake is unaffected by *C3orf58* or *SLC39A9* deficiency

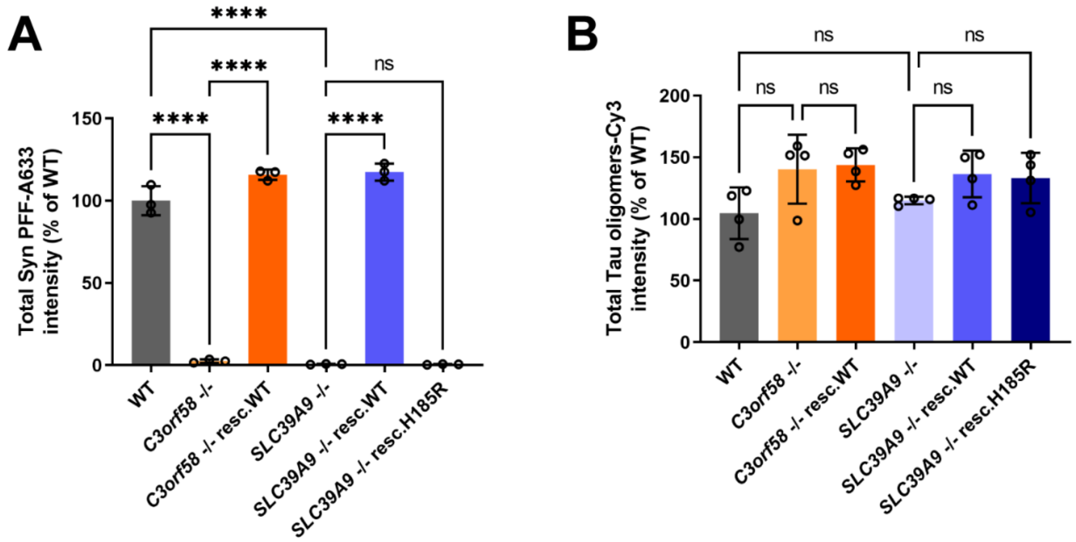

(A, B) WT, *C3orf58* -/-, *SLC39A9* -/- and indicated rescued lines were treated for 24 h with **(A)** 60 nM α-syn-PFFs-A633 (green) or **(B)** 80 nM Tau oligomers-Cy3 (magenta). After fixation and nuclei staining with Hoechst (blue), cells were imaged using a CX7 high-content microscope and PFFs-A633 or Tau oligomers-Cy3 mean total fluorescence intensity per cell were quantified with the HCS Studio Cell Analysis software. Data are shown as percent of WT values (mean ± SD). Statistical test: one-way ANOVA, * p<0.05, ** p<0.01, *** p<0.001, **** p<0.0001.

Supplementary Figure 10 – Transcriptomic analysis of *C3orf58 -/-* and *SLC39A9 -/-* RPE-1 cells reveals highly similar changes in both lines, suggesting similar modes of action

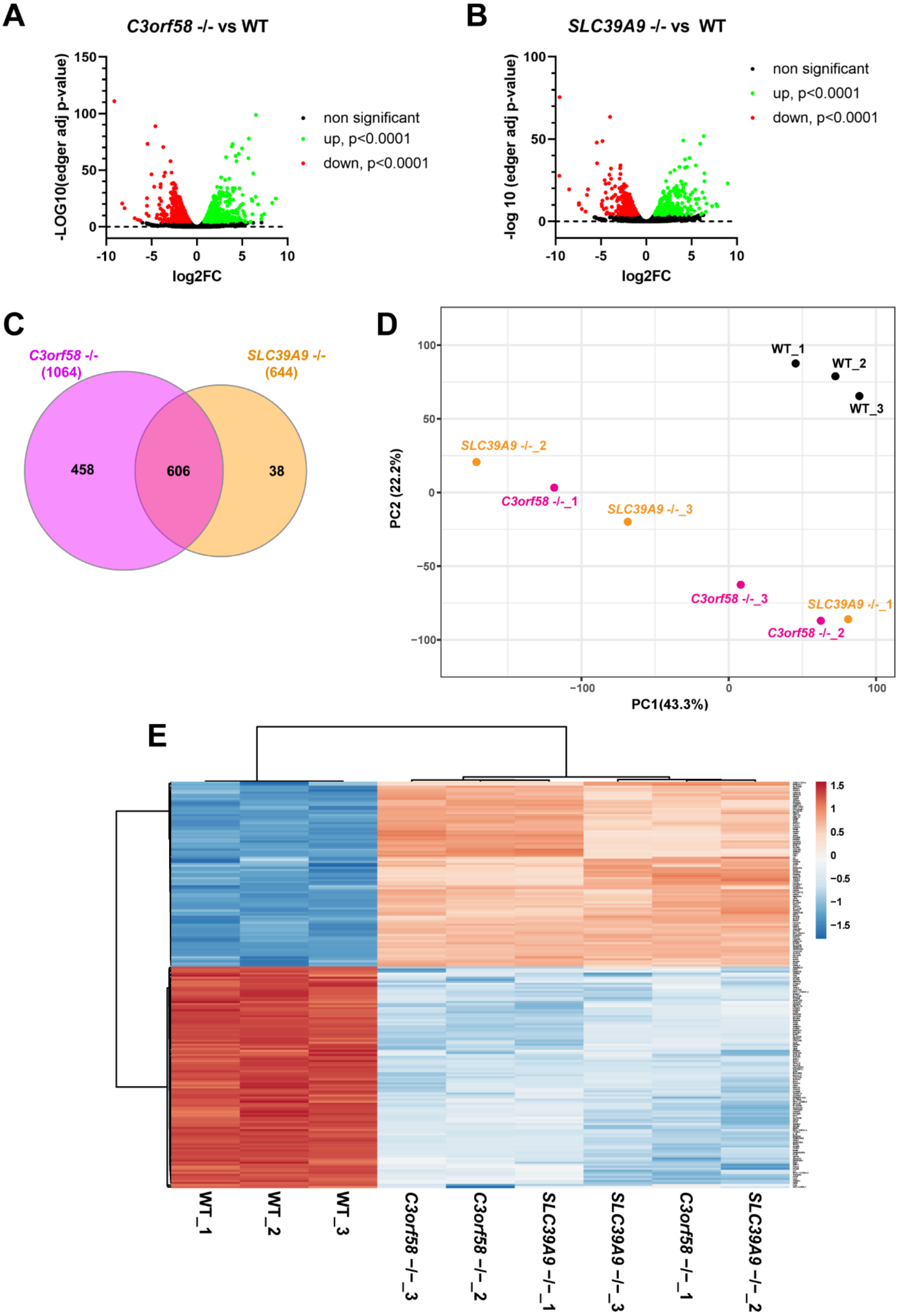

(A, B) RNAseq in *C3orf58* -/- and *SLC39A9* -/- RPE-1 cells followed by differential gene expression analysis compared to WT cells using the edgeR package (**A,** *C3orf58* -/- vs WT; **B**, *SLC39A9* -/- vs WT).

-LOG10 edgeR adjusted p-value are shown as a function of log2 fold change. Upregulated and downregulated (p<0.0001) genes are shown as green and red dots, respectively. **(C)** Venn diagram showing the high number of differentially expressed genes (p<0.0001) that are common to *C3orf58* -/- and *SLC39A9* -/- cells. **(D)** PCA analysis of gene expression values in WT, *C3orf58* -/- and *SLC39A9* -/- cells, showing that both KO cluster separately from WT, but are not sufficiently different from each other to form distinct clusters. **(E)** Heatmap from clustering analysis of the 200 most differentially expressed genes from a contrast analysis between WT samples, and all KO samples of both genotypes together. Again, the clustering groups together all KO samples, regardless of the genotype, indicative of highly similar changes in both KO lines.

Supplementary Figure 11 – KO of *C3orf58* or *SLC39A9* affect early uptake events

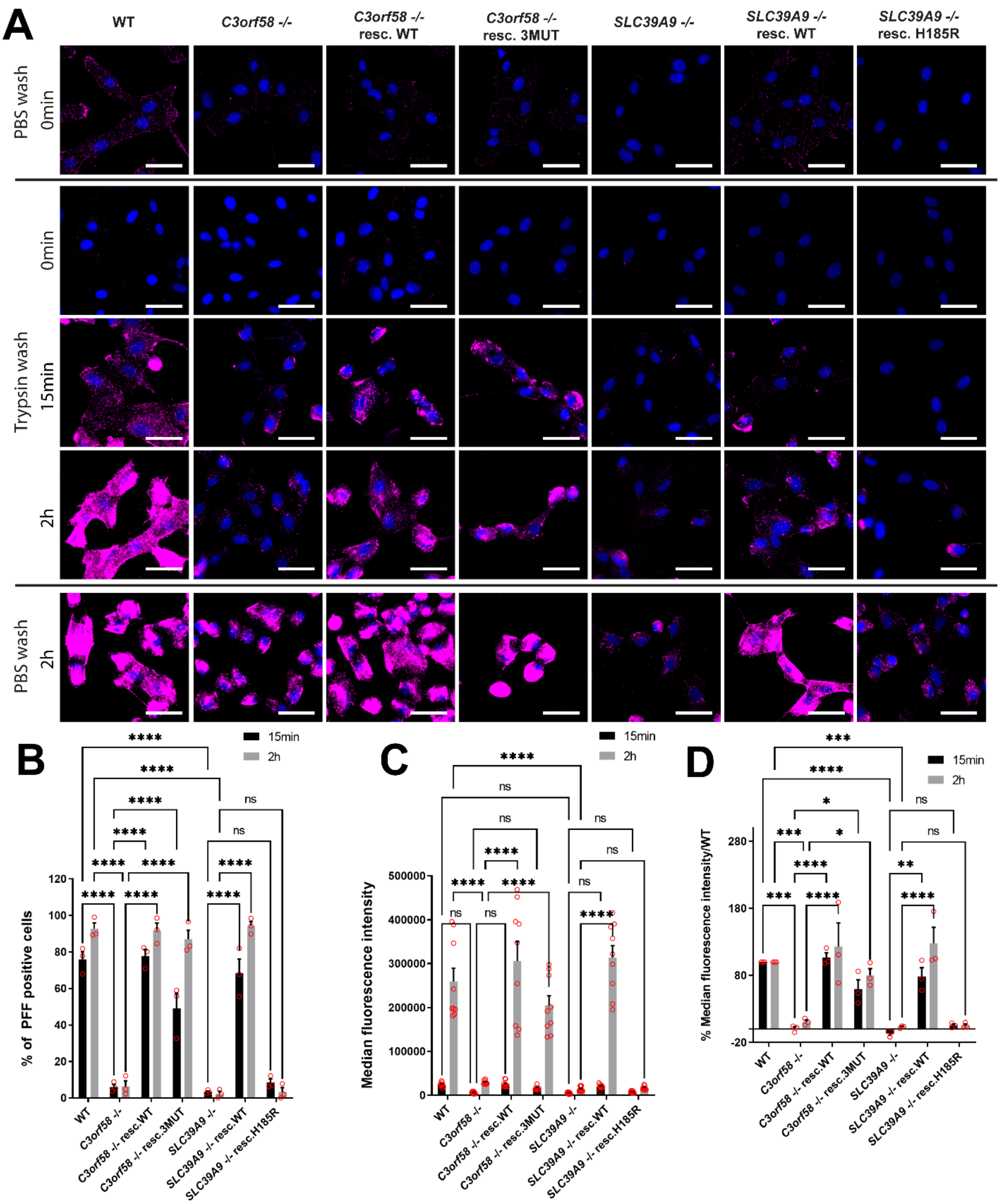

E

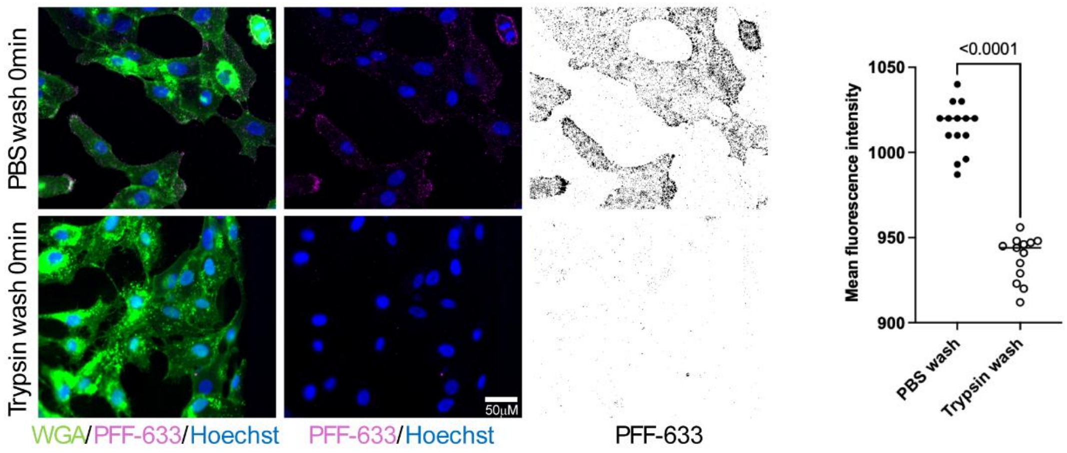

(A) RPE-1 cells of the indicated genotypes were incubated on ice with 180 nM α-syn-PFFs-A633 for 20 min in serum-free DMEM. Cells were then incubated at 37°C for the indicated times, before treatment as follows: PBS wash (top and bottom panels) or 30 sec pre-warmed trypsin wash (middle panels, to remove surface-bound PFFs and retain only signal of internalized PFFs). Cells were then fixed, and nuclei stained with Hoechst 33342 before imaging on a Zeiss epifluorescence microscope at 20x. **(B,C,D)** The indicated values were quantified using ImageJ from the experiment in **(A)**: % of PFFs positive cells (manual annotation) **(B)**, median fluorescence intensity per cell **(C)**, and relative median fluorescence intensity per cell as % of control **(D)**. Statistical tests: two-way ANOVA; * $p<0.05$; ** $p<0.01$; *** $p<0.001$; **** $p<0.0001$. n=2 independent experiments. **(E)** 30 sec trypsin wash efficiently removed extracellularly bound PFFs from the cell surface after PFFs binding at 4°C. WGA-A488 was used as a surface counterstain. The mean fluorescence intensity per cell was measured using ImageJ. Statistical test: paired t-test.

Supplementary Figure 12. Surface proteome analysis of *C3orf58* -/-, *SLC39A9* -/-, *TM9SF2* -/- and *VPS35* +/- RPE-1 cells

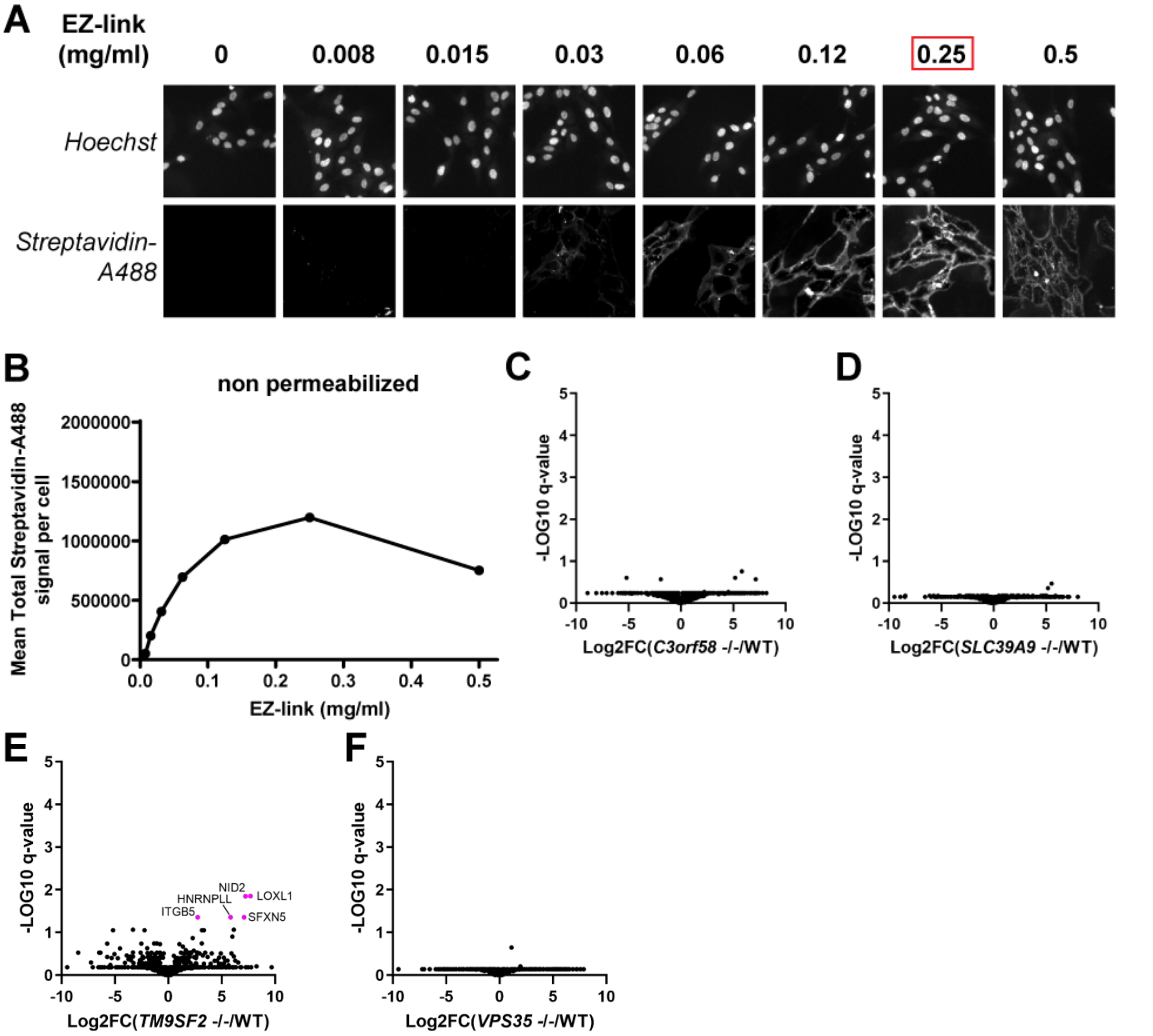

(A) RPE-1 cells were treated with increasing amounts of EZ-link biotinylation reagent for 30 min at 4°C (protocol adapted from Li et al.[6]). After quenching and fixation, nuclei were stained with Hoechst and biotinylated proteins with Alexa488-coupled Streptavidin. Images were acquired with a CX7 high-content microscope and streptavidin signals were quantified with the HCS Studio Cell Analysis software (shown in **B**). 0.25 mg/ml provided optimal biotinylation of surface proteins, and this concentration was selected for scaling up. **(C,D)** Biotinylated surface proteins from WT, *C3orf58* -/-, *SLC39A9* -/-, *TM9SF2* -/- and *VPS35* +/- cells were subjected to streptavidin pulldown followed by LC-MS/MS analysis. Changes in protein levels (estimated by peptide counts) compared to WT cells are indicated as Log2 fold-change (**C**, *C3orf58* -/-/WT; **D**, *SLC39A9* -/-/WT; **E**, *TM9SF2* -/-/WT; **E**, *VPS35* +/-/WT). Significant changes were observed solely in *TM9SF2* -/- cells (multiparametric unpaired t-tests, FDR calculated with two-stage method for Benjamini, Krieger, and Yekutieli).

Supplementary Figure 13. C3orf58 is necessary for optimal colocalization of surface-bound PFFs with surface HS

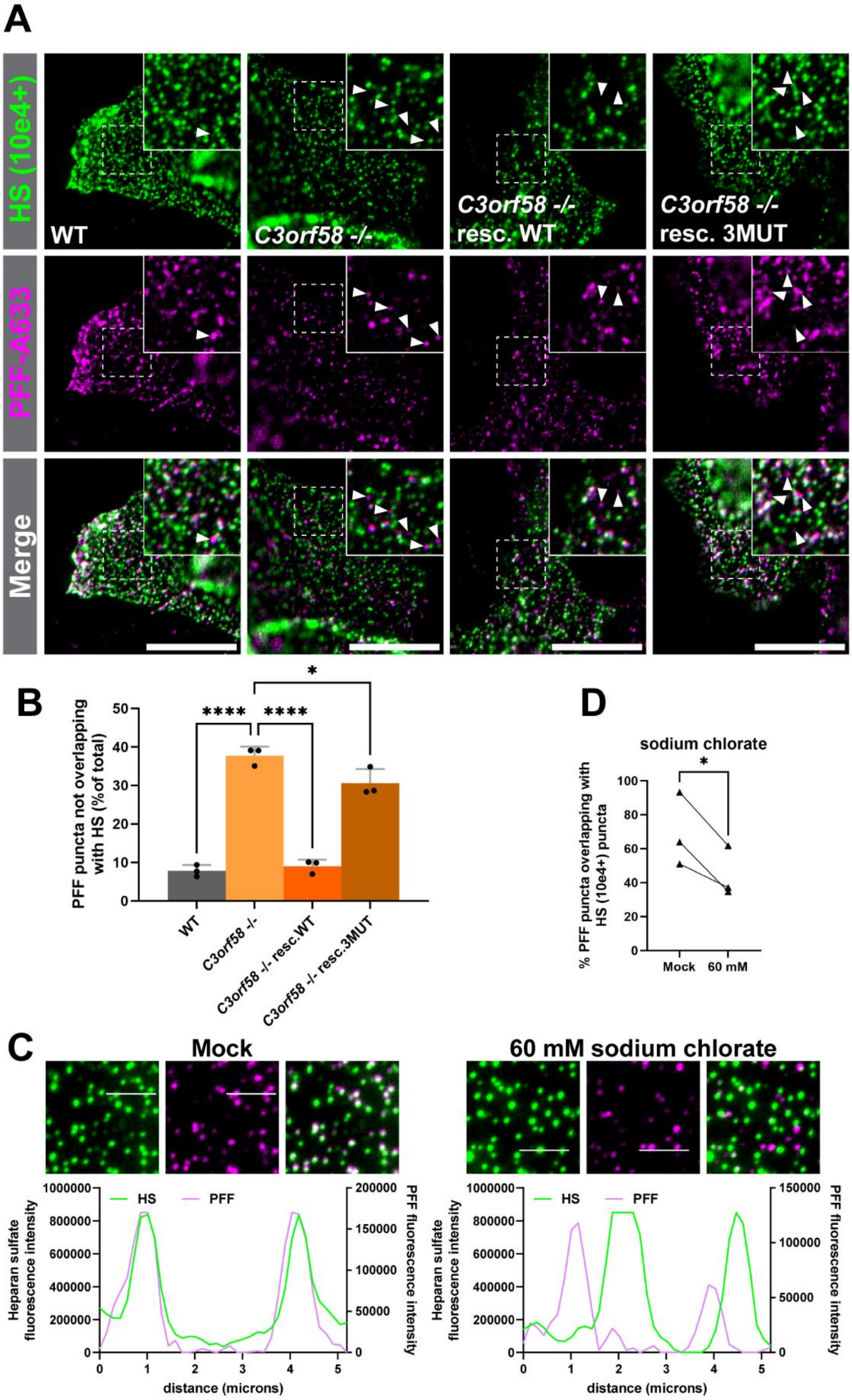

(A) PFFs-A633 binding and surface HS immunostaining were performed on ice in live WT, *C3orf58* -/- or *SLC39A9* -/- monoclonal RPE-1 cells (± indicated rescue constructs), before fixation and nuclei staining with Hoechst. Images were obtained using a Zeiss fluorescence microscope at 63x. The insets show the punctate signals for HS and PFFs at the cell surface. Scale bars: 20 µm. **(B)** The

percentage of PFFs puncta colocalizing with HS was quantified (n=3 independent experiments, >1000 dots quantified per condition, mean ±s.d. is shown). Kinase-dead C3orf58 only slightly rescued the reduced PFF/HS colocalization phenotype caused by C3orf58 deficiency, which might be due to either the importance of kinase activity or slightly lower expression level and perturbed glycosylation of this mutant compared to WT C3orf58 (**Supplementary Figure 7C**). Statistical test: one-way ANOVA; ** $p<0.01$, *** $p<0.001$, ****$p<0.0001$. **(C)** Cells from **Figure 4A,B** were imaged with a 63x objective , and the fluorescence intensity profiles along a 5.2 µm white line were measured and plotted for each set of images. **(D)** The percentage of cell surface PFFs puncta colocalized with an HS puncta (10e4 epitope) from **(C)** was quantified. N=3 independent experiments, n=698-2248 PFFs puncta counted per condition. Statistical test: paired t-test; * $p<0.05$.

Supplementary Figure 14 – mRNA levels of glycosaminoglycan related genes is affected in *C3orf58* -/- and *SLC39A9* -/- cells

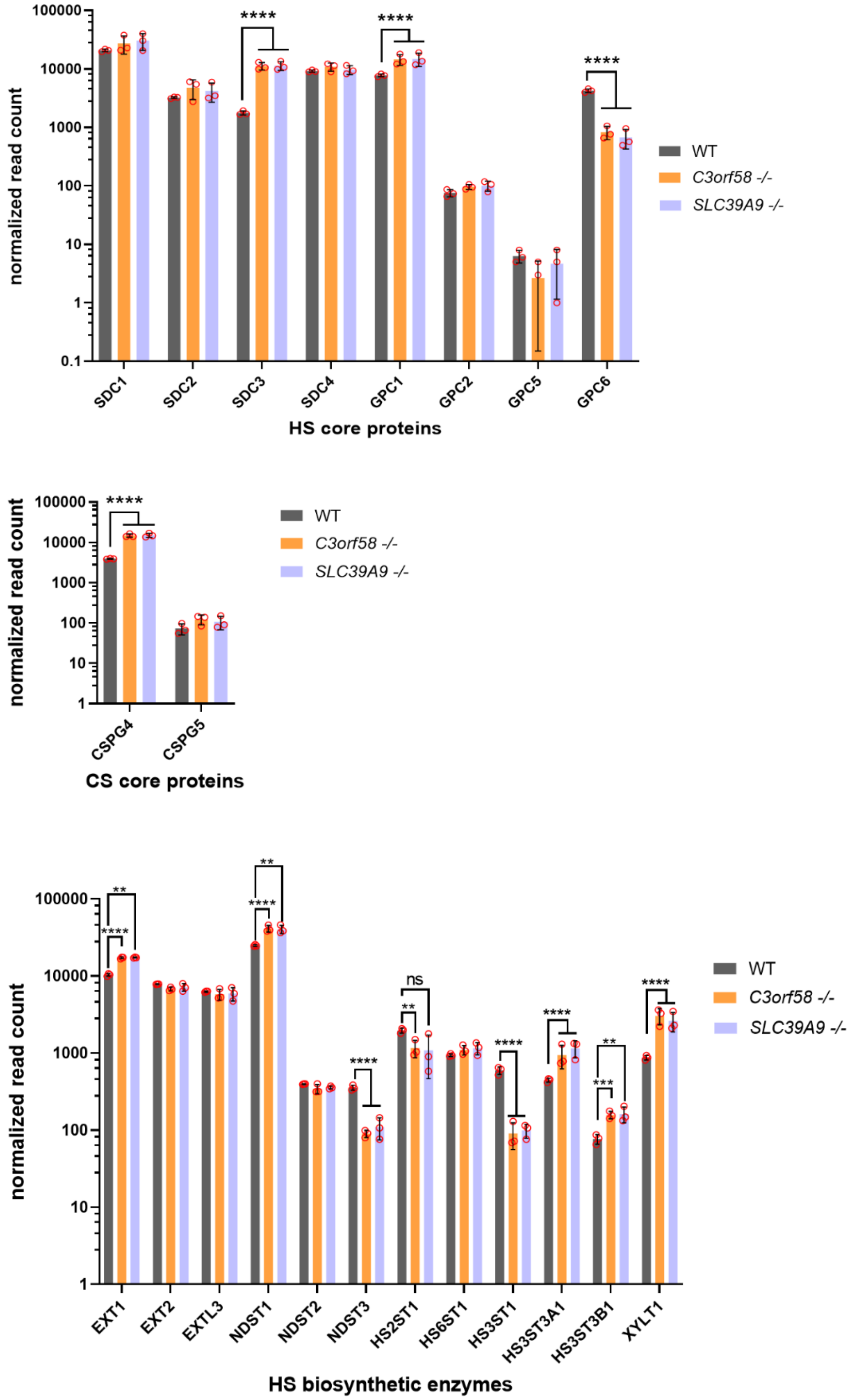

mRNA expression levels of the indicated groups of glycosaminoglycan-related genes (top, HS core proteins; bottom, HS biosynthetic enzymes; right, chondroitin sulfate (CS) core proteins). Data are expressed as mean ± SD of normalized read counts (log scale), as determined by RNA sequencing in RPE-1 cells of the indicated genotypes (WT, black; *C3orf58* -/-, dark grey; *SLC39A9* -/-, light grey). Adjusted edger p-values were calculated with the edgeR package: ** adj. p<0.01; *** adj. p<0.001; **** adj. p<0.0001.

Supplementary Figure 15. Changes in surface HS chains in *C3orf58* -/- and *SLC39A9* -/- RPE-1 cells

Di/tetra-saccharide	Abbreviation	R^1	R^2	R^3
△UA-GlcNAc	△IVA	-H	-H	--
△UA2S-GlcNAc	△IIIA	-H	$-SO_3H$	--
△UA-GlcNAc6S	△IIA	$-SO_3H$	-H	--
△UA2S-GlcNAc6S	△IA	$-SO_3H$	$-SO_3H$	--
△UA-GlcNS	△IVS	-H	-H	--
△UA2S-GlcNS	△IIIS	-H	$-SO_3H$	--
△UA-GlcNS6S	△IIS	$-SO_3H$	-H	--
△UA2S-GlcNS6S	△IS	$-SO_3H$	$-SO_3H$	--
△UA-GlcNAc6S-GlcA-GlcNS3S6S	Tetra-1	$-SO_3H$	-H	-Ac
△UA-GlcNS6S-GlcA-GlcNS3S6S	Tetra-2	$-SO_3H$	-H	$-SO_3H$
△UA-GlcNS6S-IdoA2S-GlcNS3S6S	Tetra-3	$-SO_3H$	$-SO_3H$	--
△UA-GlcNS-IdoA2S-GlcNS3S	Tetra-4	-H	-H	--
△UA2S-GlcNS-IdoA2S-GlcNS3S	Tetra-5	-H	$-SO_3H$	--

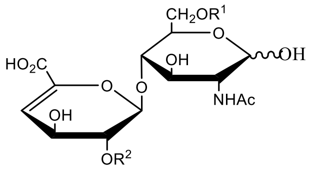

ΔIA-ΔIVA

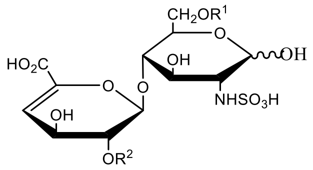

ΔIS-ΔIVS

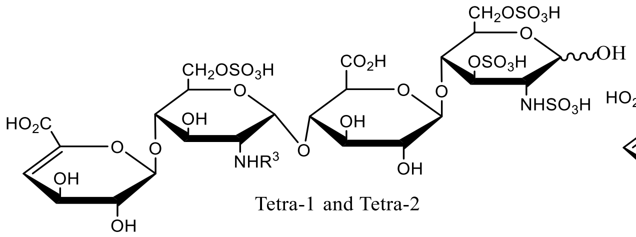

Tetra-1 and Tetra-2

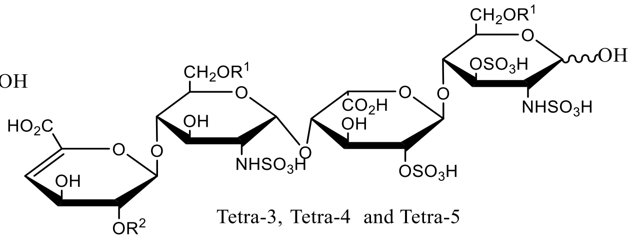

Tetra-3, Tetra-4 and Tetra-5

The HS disaccharides and tetrasaccharides analyzed in this study are described in the above table, together with the corresponding molecular structures. Complementary to Figure 7.

Supplementary Figure 15. (continued)

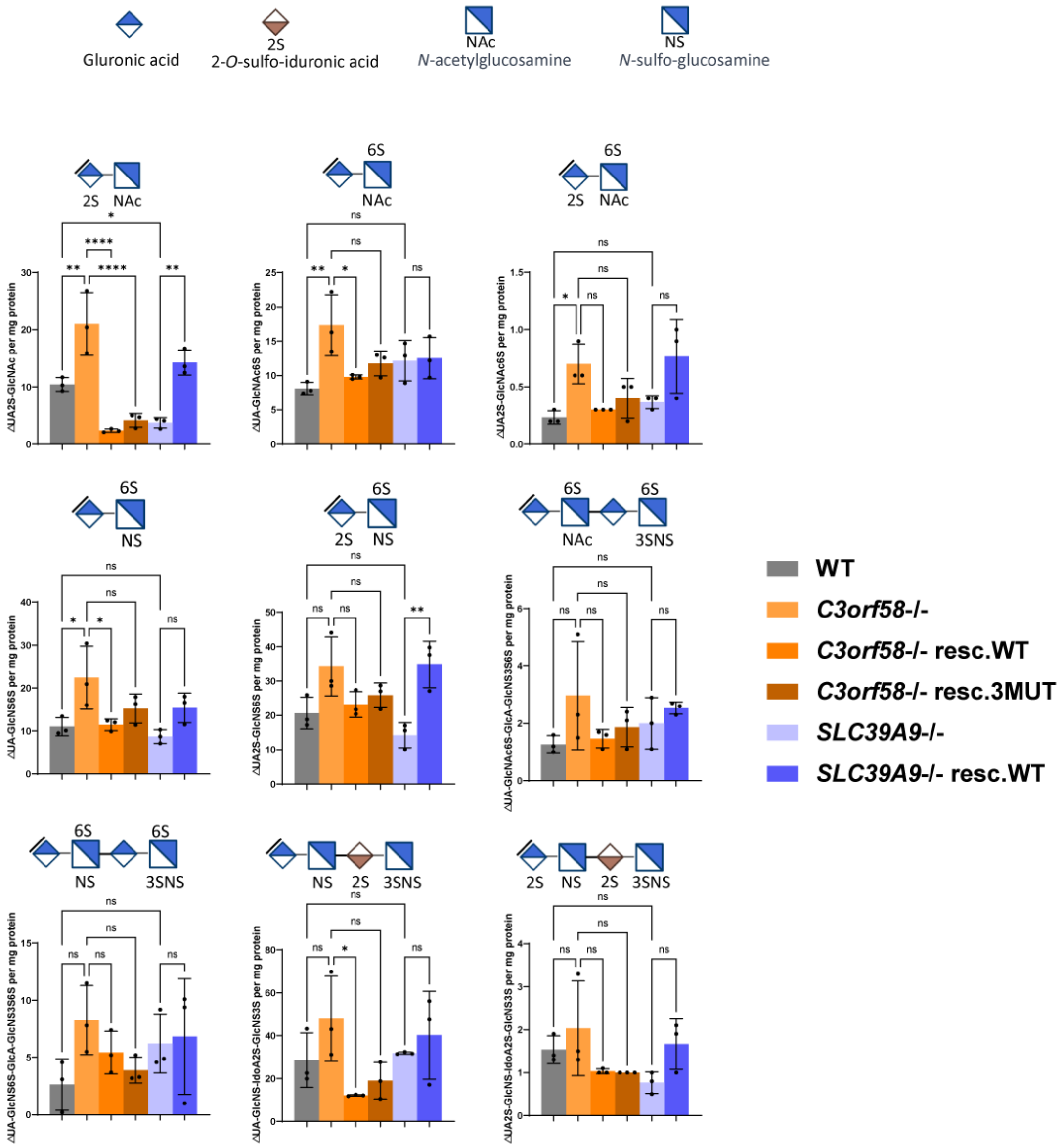

Bar graphs showing normalized quantities of the indicated HS disaccharides and tetrasaccharides isolated from the cell surface HS from WT, *C3orf58* -/- or *SLC39A9* -/- RPE-1 cells (± indicated rescue constructs). LC-MS/MS with ^{13}C-labeled internal standards was used. HS amounts were normalized to protein content from the cellular sample from which HS were isolated. A schematic representation of the analyzed species is added above each graph. Statistical tests: one-way ANOVA; * p<0.05, ** p<0.01, ****p<0.0001. Complementary to Figure 7.

Supplementary Figure 15. (continued)

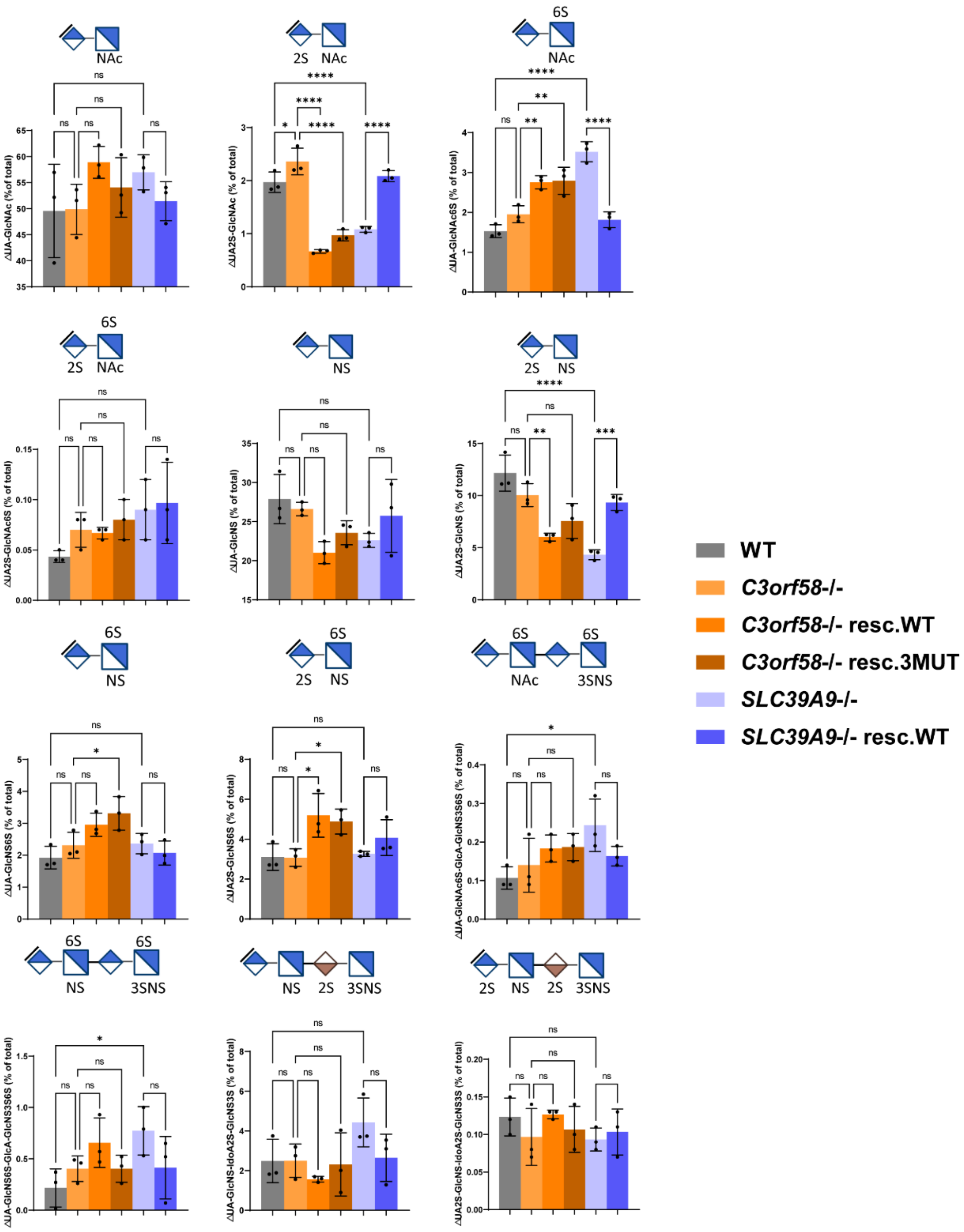

Surface HS composition (percentage of each disaccharide and tetrasaccharide species relative to total HS) of WT, *C3orf58* -/- or *SLC39A9* -/- monoclonal RPE-1 cells (± indicated rescue constructs). LC-MS/MS with ^{13}C-labeled internal standards was used. A schematic representation of the analyzed species is added above each graph. Statistical tests: one-way ANOVA; * p<0.05, ** p<0.01, *** p<0.001, ****p<0.0001. Complementary to Figure 7.

Supplementary Figure 16. Golgi structural changes in *C3orf58* -/- and *SLC39A9* -/- RPE-1 cells

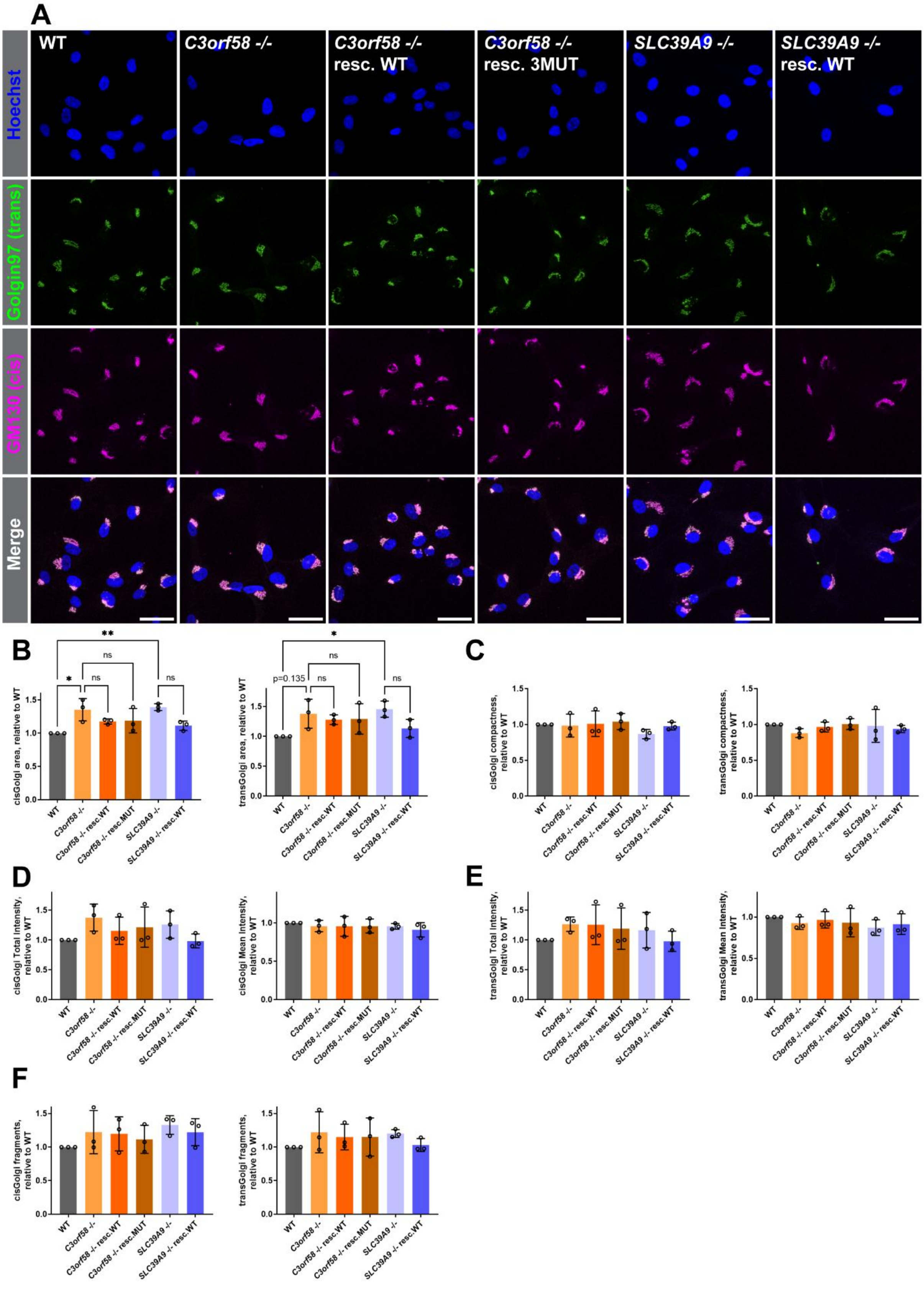

Supplementary Figure 16. (continued)

(A) WT, *C3orf58* -/- or *SLC39A9* -/- monoclonal RPE-1 cells and the indicated rescue constructs were fixed and stained by immunofluorescence for the cisGolgi marker GM130 (green) and the transGolgi marker Golgin 97 (magenta). Nuclei were stained with Hoechst. Max intensity projections of z-stacks acquired by confocal microscopy are shown. Scale bars: 50 µm. **(B-F)** A CellProfiler pipeline (adapted from[7]) was used to estimate cisGolgi and transGolgi morphology (**B**: area, **C**: compactness, **F**: number of fragments) and measure signal intensity (**D**: mean total intensity, **E**: mean average intensity) of both markers. Data are presented as mean ± SD. Statistical test: one-way ANOVA; * $p<0.05$, ** $p<0.01$.

Supplementary Figure 17. Generation of *C3orf58* -/- and *SLC39A9* -/- iPSCs

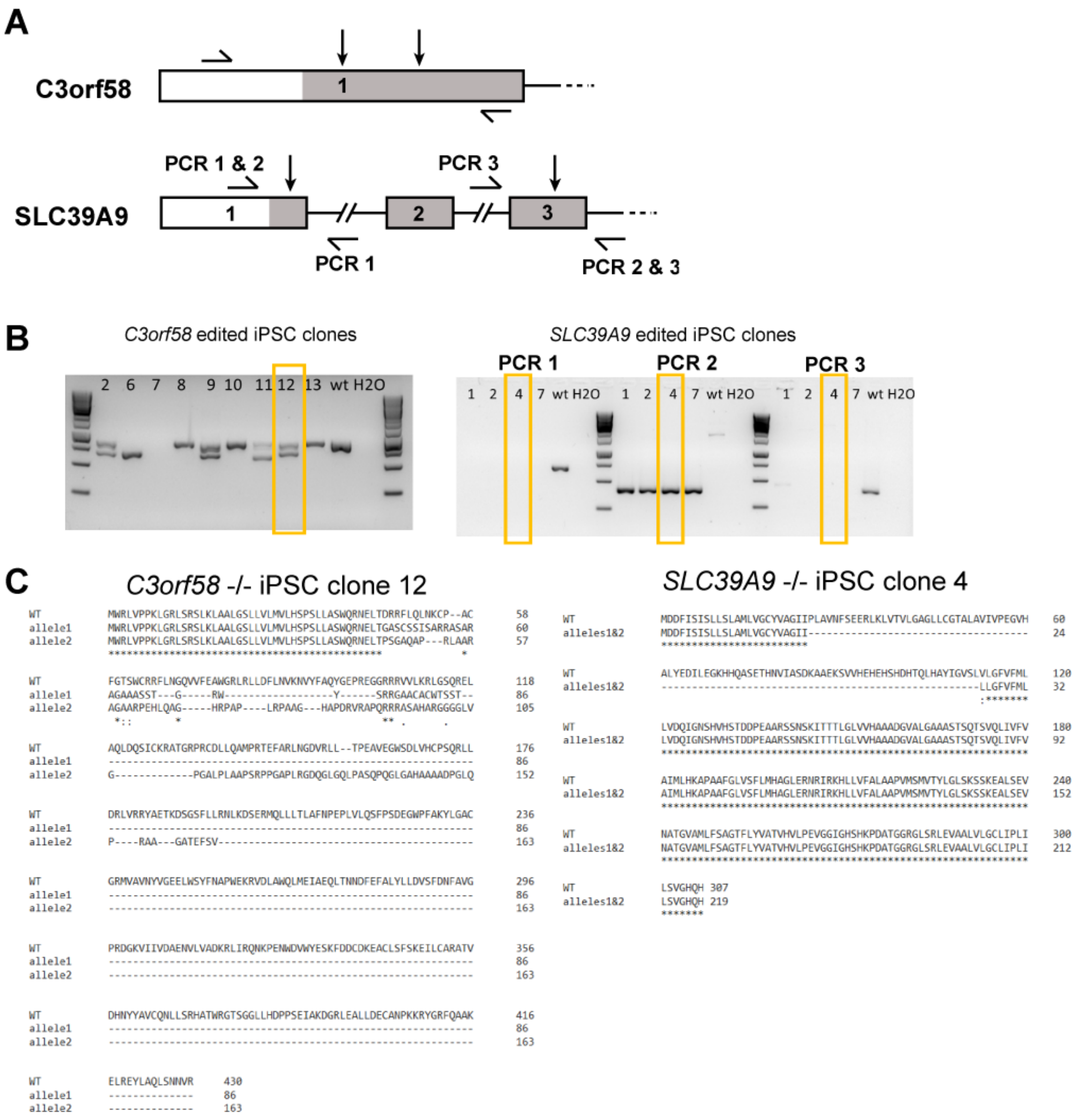

(A) Schematic representation of KO and PCR-screening strategies for the *C3orf58* and *SLC39A9* genes in human iPSCs. **(B)** Allelic sequences from the indicated CRISPR-edited iPSC clones were PCR-amplified using Q5 polymerase and oligonucleotides indicated in **(A)**. Orange boxes indicate selected clones. **(C)** PCR products from **(B)** were subjected to Sanger sequencing, and the obtained allelic sequences were translated *in silico* before alignment on the WT sequence. The identified allelic sequences are shown and aligned to the reference WT sequence. For *C3orf58* -/- clone 12, alleles 1 and 2 presented cumulated 20 bp (16 bp + 4 bp) or uninterrupted 179 bp deletions respectively, resulting in a frameshift and premature stop codons. For *SLC39A9* -/- clone 4 iPSCs, a homozygous deletion (264 coding bp) resulted in the loss of 88 amino acids from the remaining protein product (identical to allele 2 from *SLC39A9* -/- clone 10 RPE-1 cells used throughout the study, see Supplementary Figure 5C), which inactivates the Zn^{2+} transporting function (Supplementary Figure 8).

Supplementary Figure 17. (continued)

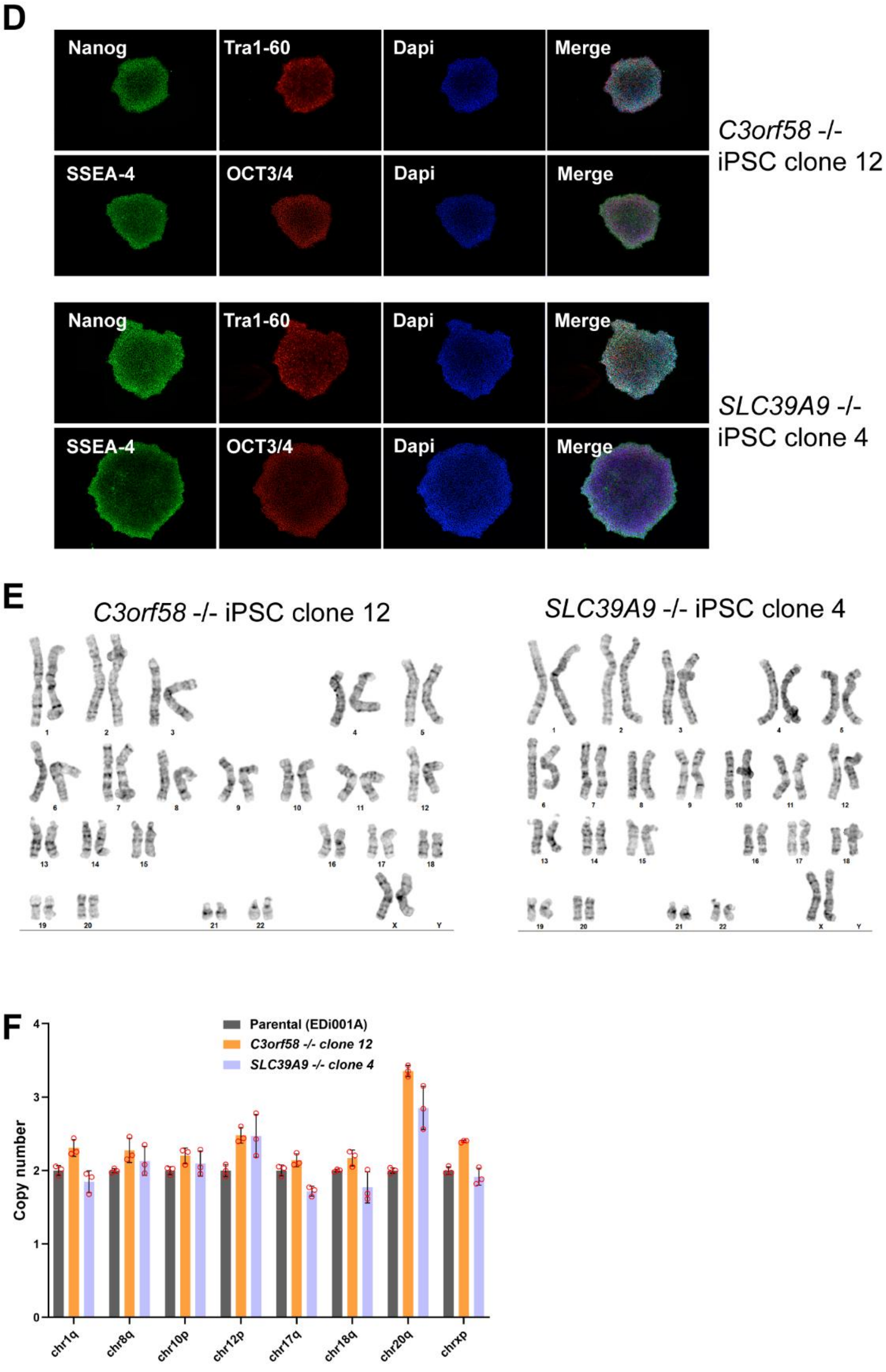

(D) Pluripotency of both edited iPSC lines was verified by immunostaining against the pluripotency markers Nanog, Tra1-60, SSEA-4, and OCT3/4. **(E)** Karyotype of *C3orf58* -/- and *SLC39A9* -/- monoclonal iPSCs. **(F)** CRISPR-edited iPSC showed a duplication of one copy of the chr20q region as assessed by a qPCR-based genome stability test, but this did not prevent proper differentiation into iMGL (see Supplementary Figure 18).

Supplementary Figure 18. Human microglia derived from *C3orf58* -/- and *SLC39A9* -/- iPSC correctly express microglial markers

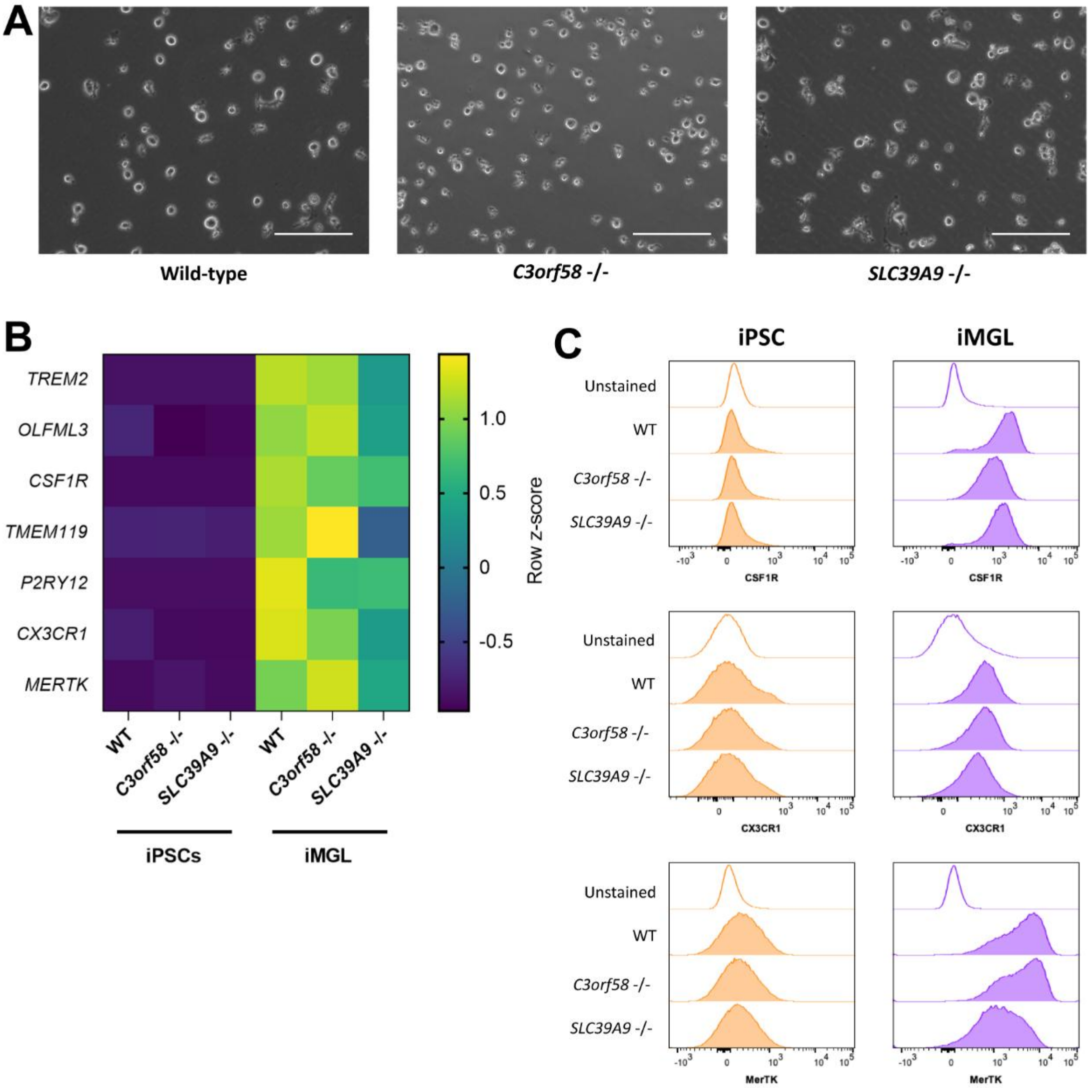

(A) Representative bright field images of WT, *C3orf58* -/- and *SLC39A9* -/- human iPSC-derived microglia (iMGL) used in the study. Scale bars: 150 µm. **(B)** qRT-PCR was used to measure mRNA levels of the indicated microglial marker genes in iPSCs or iMGL of the indicated genotypes. Levels were normalized to *GAPDH* and *YWHAZ* genes. iPSC n=4, iMGL n=3. **(C)** Surface microglial markers CSF1R, CX3CR1, and MerTK expression levels were assessed by flow cytometry in iPSC or iMGL of the indicated genotypes.

Supplementary Figure 19 – α-syn PFFs uptake is partially mediated by macropinocytosis in RPE-1 cells

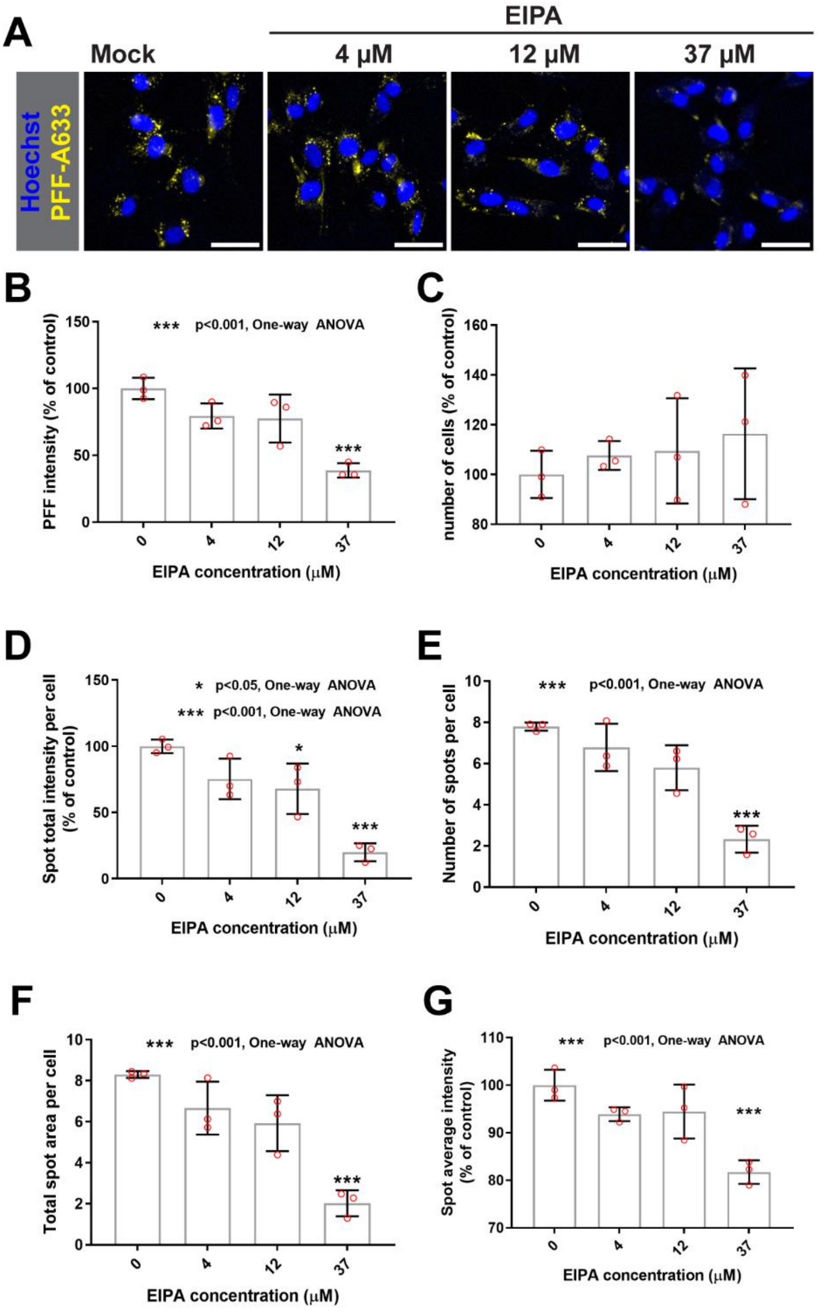

(A) RPE-1 cells were treated with increasing concentrations of the macropinocytosis inhibitor EIPA for 30 min at 37°C before 14h overnight treatment with 60 nM PFFs-A633, fixation, nuclei staining with Hoechst, imaging and **(B-G)** quantification with a CX7 high-content microscope. Scale bars: 30 µm. Parameters quantified are shown as mean ± SD: **(B)** total PFFs intensity, **(C)** cell number, **(D)** total PFFs spots intensity per cell, **(E)** number of PFFs spots per cell, **(F)** area covered by PFFs spots per cell, and **(G)** average intensity within PFFs spots. 37 µM treatment resulted in a significant decrease in PFFs uptake.

Supplementary Figure 20 – Expression of dox-inducible NDST1-HA by Western blot

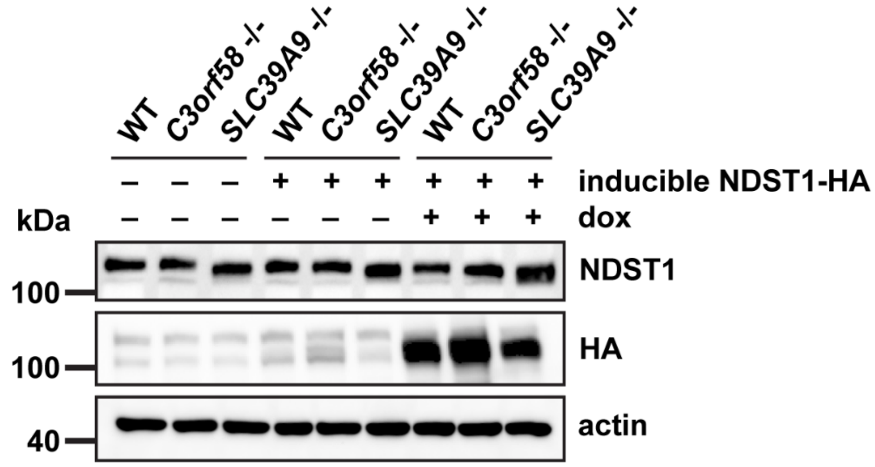

WT, *C3orf58* -/- and *SLC39A9* -/-RPE-1 cells were transduced or not with a lentivirus driving expression of doxycycline (dox) inducible NDST1-HA, and treated with or without 25ng/ml doxycycline for 96 hours before processing for Western blot analysis.

Supplementary Figure 21 – Uncropped and unedited blots images

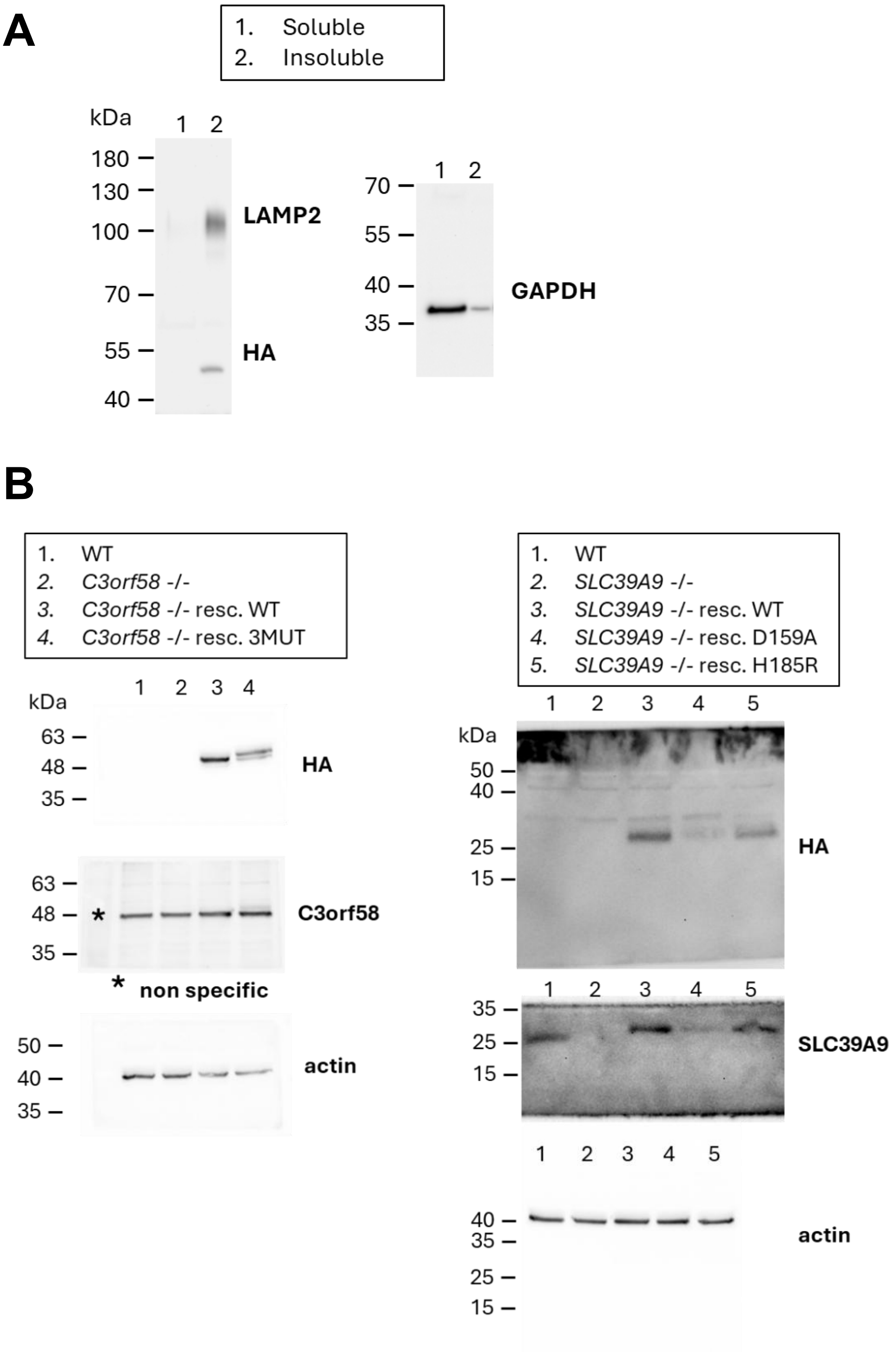

C

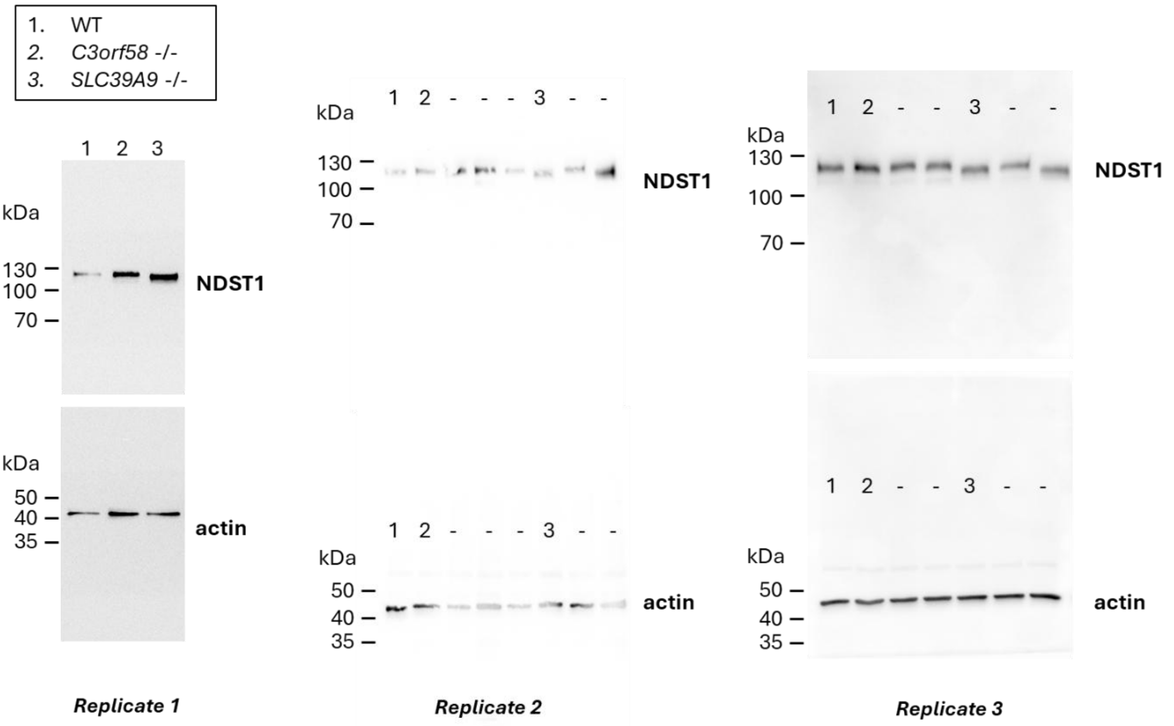

D

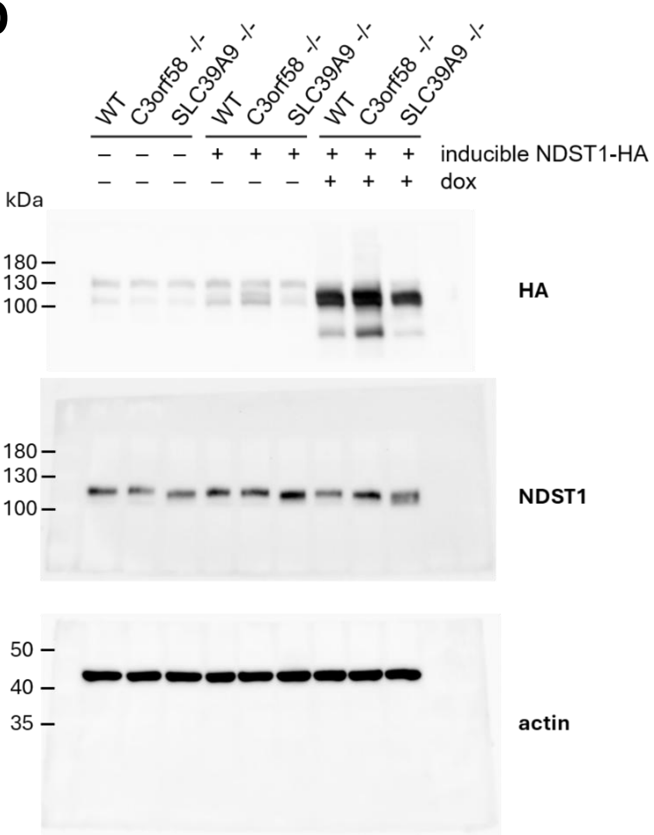

E

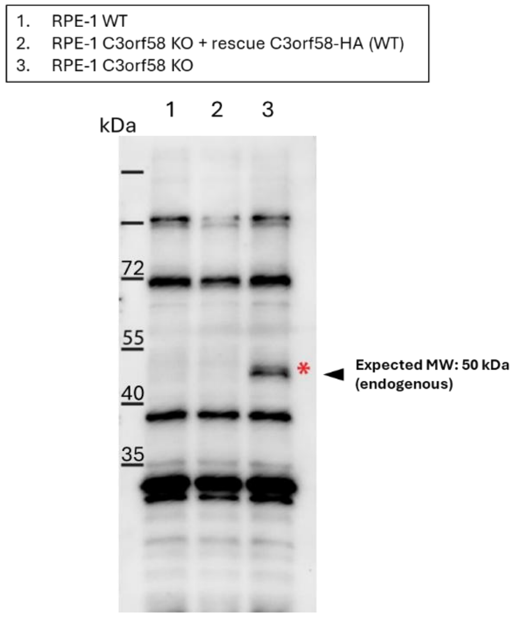

(A) Full blots relative to Supplementary Figure 7A, **(B)** Supplementary Figure 7C, **(C)** Figure 8A, and **(D)** Supplementary Figure 20. **(E)** A home-made C3orf58-specific antibody failed to detect endogenous C3orf58, but allowed to clearly establish that WT C3orf58-HA (resc. WT line) is strongly overexpressed compared to endogenous C3orf58.

Supplementary Figure 22 – Gating strategies for FACS experiments

A

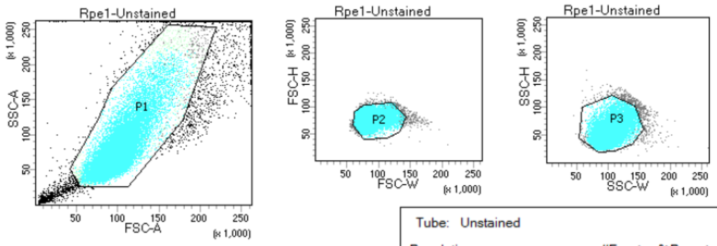

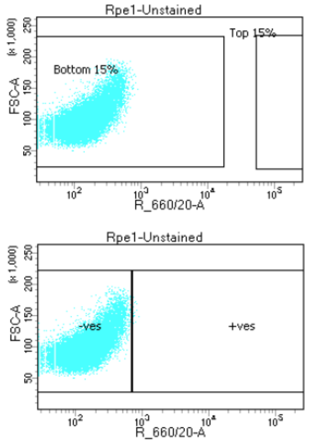

Tube: Unstained

Population	#Events	%Parent	%Total
All Events	37,271	####	100.0
P1	34,436	92.4	92.4
P2	33,918	98.5	91.0
P3	33,788	98.1	90.7
P2 AND P3	33,521	97.3	89.9
+ves	17	0.1	0.0
-ves	33,510	100.0	89.9
Top 15%	0	0.0	0.0
Bottom 15%	33,521	100.0	89.9

Experiment Name: Nishani September 11 2017
Specimen Name: Rpe1
Tube Name: Unstained
Record Date: Sep 11, 2017 11:12:31 AM
$OP: Administrator

Population	#Events	%Parent	R_660/20-A Median	R_660/20-A SD	R_660/20-A %CV
All Events	37,271	####	138	1,078	583.7
P1	34,436	92.4	135	100	61.9
P2	33,918	98.5	135	96	60.1
P3	33,788	98.1	133	91	58.2
P2 AND P3	33,521	97.3	133	90	57.7
+ves	17	0.1	722	47	6.4
-ves	33,510	100.0	133	90	57.4
Top 15%	0	0.0	####	####	####
Bottom 15%	33,521	100.0	133	90	57.7

B

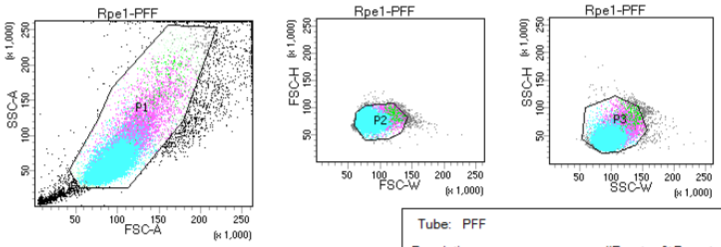

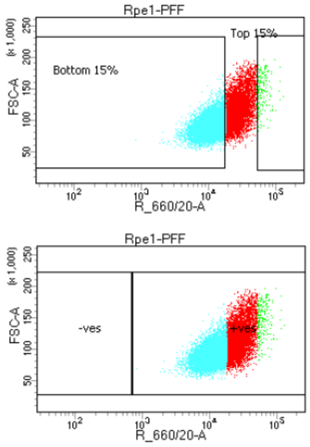

Tube: PFF

Population	#Events	%Parent	%Total
All Events	31,610	####	100.0
P1	29,023	91.8	91.8
P2	28,587	98.5	90.4
P3	28,518	98.3	90.2
P2 AND P3	28,303	97.5	89.5
+ves	28,303	100.0	89.5
-ves	0	0.0	0.0
Top 15%	231	0.8	0.7
Bottom 15%	19,639	69.4	62.1

Experiment Name: Nishani September 11 2017
Specimen Name: Rpe1
Tube Name: PFF
Record Date: Sep 11, 2017 11:14:00 AM
$OP: Administrator

Population	#Events	%Parent	R_660/20-A Median	R_660/20-A SD	R_660/20-A %CV
All Events	31,610	####	14,186	17,000	91.0
P1	29,023	91.8	13,949	10,350	61.8
P2	28,587	98.5	13,899	9,868	59.8
P3	28,518	98.3	13,879	9,431	57.6
P2 AND P3	28,303	97.5	13,863	9,315	57.1
+ves	28,303	100.0	13,863	9,315	57.1
-ves	0	0.0	####	####	####
Top 15%	231	0.8	60,717	13,208	20.4
Bottom 15%	19,639	69.4	11,571	3,262	28.0

C

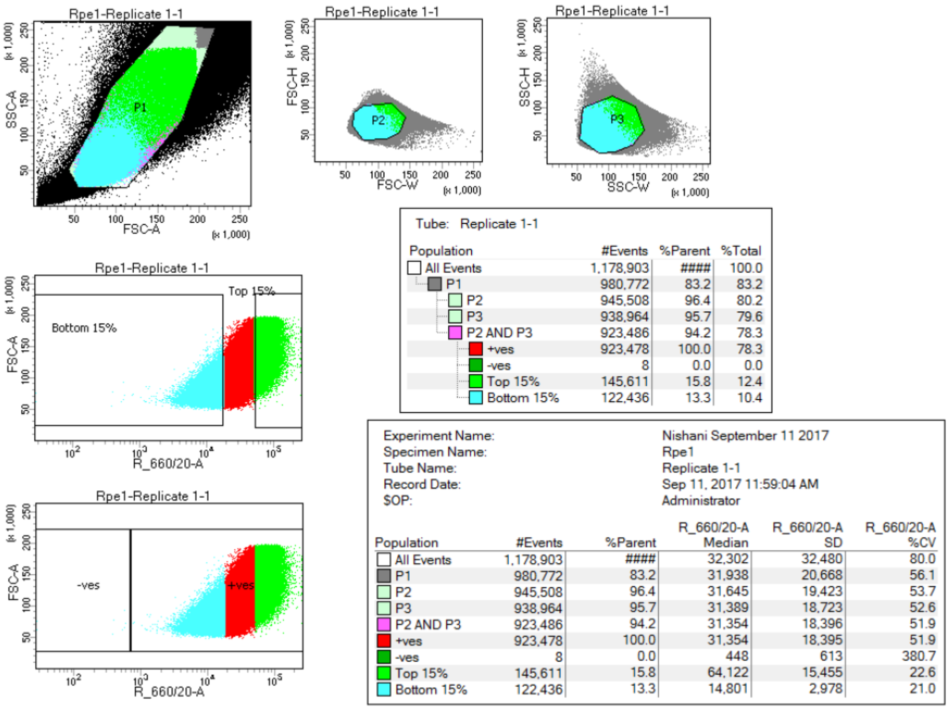

Tube: Replicate 1-1

Population	#Events	%Parent	%Total
All Events	1,178,903	####	100.0
P1	980,772	83.2	83.2
P2	945,508	96.4	80.2
P3	938,964	95.7	79.6
P2 AND P3	923,486	94.2	78.3
+ves	923,478	100.0	78.3
-ves	8	0.0	0.0
Top 15%	145,611	15.8	12.4
Bottom 15%	122,436	13.3	10.4

Experiment Name: Nishani September 11 2017
Specimen Name: Rpe1
Tube Name: Replicate 1-1
Record Date: Sep 11, 2017 11:59:04 AM
$OP: Administrator

Population	#Events	%Parent	R_660/20-A Median	R_660/20-A SD	R_660/20-A %CV
All Events	1,178,903	####	32,302	32,480	80.0
P1	980,772	83.2	31,938	20,668	56.1
P2	945,508	96.4	31,645	19,423	53.7
P3	938,964	95.7	31,389	18,723	52.6
P2 AND P3	923,486	94.2	31,354	18,396	51.9
+ves	923,478	100.0	31,354	18,395	51.9
-ves	8	0.0	448	613	380.7
Top 15%	145,611	15.8	64,122	15,455	22.6
Bottom 15%	122,436	13.3	14,801	2,978	21.0

D

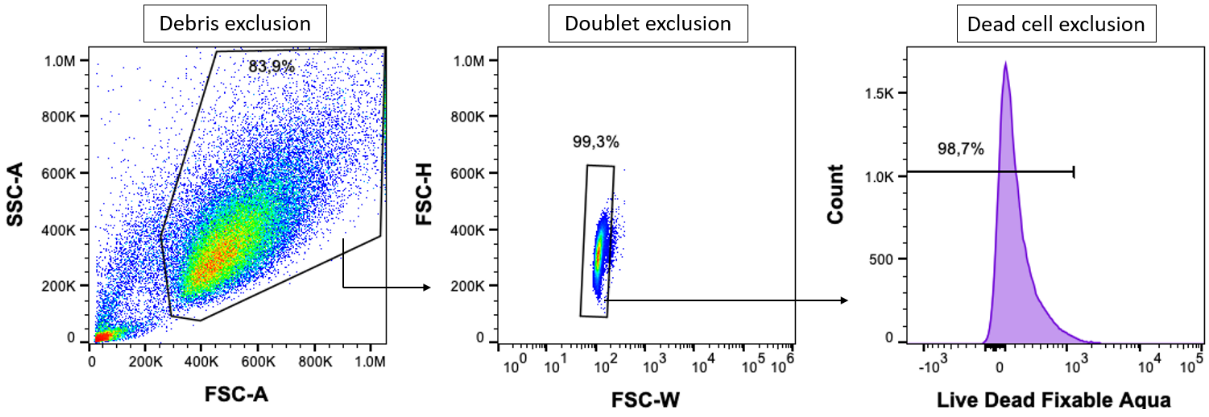

(A,B) Gating strategies used to define PFF positive and negative cells using untreated **(A)** or 15 nM PFFs-A633 treated **(B)** RPE-1 cells. **(C)** From the screen cell population, the top 15 % and bottom 15 % cells based on PFFs-A633 fluorescence intensity. Replicate 1 from the screen is shown. **(D)** Gating strategy for analyzing iPSC-derived microglial populations.

Supplementary References

1. Senturk, E. & Manfredi, J. J. p53 and cell cycle effects after DNA damage. *Methods Mol Biol* **962**, 49–61 (2013).
2. Gao, Q. *et al.* CDKN1A promotes Cis-induced AKI by inducing cytoplasmic ROS production and ferroptosis. *Food and Chemical Toxicology* **193**, 115003 (2024).
3. Chen, T., Sun, Y., Ji, P., Kopetz, S. & Zhang, W. Topoisomerase IIα in Chromosome Instability and Personalized Cancer Therapy. *Oncogene* **34**, 4019–4031 (2015).
4. Frugoni, F. *et al.* A Novel Mutation in the POLE2 Gene Causing Combined Immunodeficiency. *J Allergy Clin Immunol* **137**, 635-638.e1 (2016).
5. Haapaniemi, E., Botla, S., Persson, J., Schmierer, B. & Taipale, J. CRISPR–Cas9 genome editing induces a p53-mediated DNA damage response. *Nature Medicine* **24**, 927 (2018).
6. Li, Y. *et al.* Sensitive profiling of cell surface proteome by using an optimized biotinylation method. *Journal of Proteomics* **196**, 33–41 (2019).
7. Mejia, I., Chen, Y.-C. & Díaz, B. Analysis of Golgi Morphology Using Immunofluorescence and CellProfiler Software. *Methods Mol Biol* **2557**, 765–784 (2023).
