# Supplementary material for: Novel regulators of heparan sulfate proteoglycans modulate cellular uptake of α-synuclein fibrils
Source: Commun Biol. 2025 Oct 6;8:1426. doi: 10.1038/s42003-025-08786-2 (PMC12501064; doi:10.1038/s42003-025-08786-2)
Supplement: Supplementary file 5 — Reporting Summary [file 42003_2025_8786_MOESM5_ESM.pdf]

nature portfolio

Corresponding author(s): DAPR COMMSBIO-24-8909

Last updated by author(s): Jul 30, 2025

# Reporting Summary

Nature Portfolio wishes to improve the reproducibility of the work that we publish. This form provides structure for consistency and transparency in reporting. For further information on Nature Portfolio policies, see our Editorial Policies and the Editorial Policy Checklist.

## Statistics

For all statistical analyses, confirm that the following items are present in the figure legend, table legend, main text, or Methods section.

| n/a | Confirmed | |
|---|---|---|
| ☐ | ☒ | The exact sample size (*n*) for each experimental group/condition, given as a discrete number and unit of measurement |
| ☐ | ☒ | A statement on whether measurements were taken from distinct samples or whether the same sample was measured repeatedly |
| ☐ | ☒ | The statistical test(s) used AND whether they are one- or two-sided<br>*Only common tests should be described solely by name; describe more complex techniques in the Methods section.* |
| ☒ | ☐ | A description of all covariates tested |
| ☐ | ☒ | A description of any assumptions or corrections, such as tests of normality and adjustment for multiple comparisons |
| ☐ | ☒ | A full description of the statistical parameters including central tendency (e.g. means) or other basic estimates (e.g. regression coefficient) AND variation (e.g. standard deviation) or associated estimates of uncertainty (e.g. confidence intervals) |
| ☒ | ☐ | For null hypothesis testing, the test statistic (e.g. *F*, *t*, *r*) with confidence intervals, effect sizes, degrees of freedom and *P* value noted<br>*Give P values as exact values whenever suitable.* |
| ☒ | ☐ | For Bayesian analysis, information on the choice of priors and Markov chain Monte Carlo settings |
| ☒ | ☐ | For hierarchical and complex designs, identification of the appropriate level for tests and full reporting of outcomes |
| ☒ | ☐ | Estimates of effect sizes (e.g. Cohen's *d*, Pearson's *r*), indicating how they were calculated |

*Our web collection on statistics for biologists contains articles on many of the points above.*

## Software and code

Policy information about availability of computer code

| | |
|---|---|
| Data collection | Softwares used for microscopy images collection include ZEN 3.0 blue edition (Zeiss epifluorescence microscope), HCS Studio Cell Analysis v6.6.1 (CX7 high-content microscope), Harmony v.5.2 (Opera Phoenix microscope), and NIS-Elements AR v5.42.03 (Nikon A1 confocal microscope). For acquisition of ThT fluorescence data with the FLUOstar Omega, the Reader Control software was used. For FACS data acquisition, BD FACSDiva v8.0 was used for BD Biosciences FACS Aria II cell sorter, BD FACS™ Sorftware v1.2.0.142 for BD Biosciences Influx Cell sorter, and Attune Cytometric Software v6 for Attune Nxt Flow Cytometer. |
| Data analysis | For analysis of NGS files from the CRISPR screen, Mageck v0.5.6 was used. For high-content microscopy images analysis, the HCS Studio Cell Analysis (CX7 microscope) or Harmony v5.2 (Opera Phoenix) softwares were used. For analysis of epifluorescence microscopy images, ImageJ v1.53t was used. For analysis of Nikon A1-acquired images, CellProfiler v4.2.5 was used. FLowJo v10.4 was used for FACS analysis. For protein identification by MS, Raw MS files were analyzed using the Mascot search engine through the iProphet pipeline integrated into Prohits. For downstream proteomics data analysis, ProHits was used. For data processing of LC-MS/MS analysis of HS, TraceFinder was used. RNAseq analysis was performed using the GenPipes RNAseq analysis pipeline version 3.0 run on Compute Canada's high-performance computing cluster. For RT-qPCR analysis, the QuantStudio Design & Analysis Software was used. To generate graphs and perform statistical tests not included in pipelines described above, GraphPad Prism 9.0 was used. |

For manuscripts utilizing custom algorithms or software that are central to the research but not yet described in published literature, software must be made available to editors and reviewers. We strongly encourage code deposition in a community repository (e.g. GitHub). See the Nature Portfolio guidelines for submitting code & software for further information.

# Data

Policy information about availability of data

All manuscripts must include a data availability statement. This statement should provide the following information, where applicable:

- Accession codes, unique identifiers, or web links for publicly available datasets
- A description of any restrictions on data availability
- For clinical datasets or third party data, please ensure that the statement adheres to our policy

All source data underlying the graphs and charts presented in the main and supplementary figures are available in Supplementary Data 1. The mass spectrometry proteomics data have been deposited to the ProteomeXchange Consortium via the PRIDE90 partner repository with the dataset identifier PXD065224. RNAseq data have been deposited to the Gene Expression Omnibus (accession number: GSE299483). All other data types are available on request (including but not limited to microscopy images, Sanger sequencing ab1 files, FACS data, and scripts).

# Research involving human participants, their data, or biological material

Policy information about studies with human participants or human data. See also policy information about sex, gender (identity/presentation), and sexual orientation and race, ethnicity and racism.

| | |
|---|---|
| Reporting on sex and gender | n.a. |
| Reporting on race, ethnicity, or other socially relevant groupings | n.a. |
| Population characteristics | n.a. |
| Recruitment | n.a. |
| Ethics oversight | n.a. |

Note that full information on the approval of the study protocol must also be provided in the manuscript.

# Field-specific reporting

Please select the one below that is the best fit for your research. If you are not sure, read the appropriate sections before making your selection.

☒ Life sciences ☐ Behavioural & social sciences ☐ Ecological, evolutionary & environmental sciences

For a reference copy of the document with all sections, see nature.com/documents/nr-reporting-summary-flat.pdf

# Life sciences study design

All studies must disclose on these points even when the disclosure is negative.

| | |
|---|---|
| Sample size | No sample size calculation was performed. For the screen, the number of cells sorted for each sample was defined to reach at least 100-fold library coverage (138-fold was reached). For high-content microscopy analyses of RPE-1 cells, a minimum of 9 fields of view was analyzed, allowing analysis of a minimum of 34 cells in some very rare instances, although typically, most samples contained >500 cells. For other cell-based microscopy analyses (confocal, epifluorescence), a minimum of 28 cells per replicate were analyzed. For all types of analyses except the screen, a minimum of 3 replicates samples per experimental group were used, wehere replicates were defind as independently prepared biological replicates. These numbers are in accordance with the standards of the field. |
| Data exclusions | No data was excluded from the analyses. |
| Replication | All data presented was extracted from a minimum of 3 independent experiments, except in the CRISPR screen (duplicate, widely accepted in the field of CRISPR screening). |
| Randomization | Samples were grouped manually. However, all samples within a given experiment were analyzed in an unbiased manner via the use of identical analyses pipelines between samples. Thus, our manual grouping had no influence on the results. |
| Blinding | No blinding was performed, but in cases where this could be an issue (e.g. acquisition of microscopy images), random fields were acquired to prevent bias. |

# Reporting for specific materials, systems and methods

We require information from authors about some types of materials, experimental systems and methods used in many studies. Here, indicate whether each material, system or method listed is relevant to your study. If you are not sure if a list item applies to your research, read the appropriate section before selecting a response.

## Materials & experimental systems

| n/a | Involved in the study |
|---|---|
| ☐ | ☒ Antibodies |
| ☐ | ☒ Eukaryotic cell lines |
| ☒ | ☐ Palaeontology and archaeology |
| ☒ | ☐ Animals and other organisms |
| ☒ | ☐ Clinical data |
| ☒ | ☐ Dual use research of concern |
| ☒ | ☐ Plants |

## Methods

| n/a | Involved in the study |
|---|---|
| ☒ | ☐ ChIP-seq |
| ☐ | ☒ Flow cytometry |
| ☒ | ☐ MRI-based neuroimaging |

# Antibodies

Antibodies used

Complete information available in Supplementary Table S6
*target/company/cat #
*Heparan sulfate (10e4)/AMSbio/370255-1
*HA tag/Biolegend/902301
*HA tag/Cell Signaling/2367S
*GM130/BD Biosciences/610823
*Golgin 97/Cell Signaling/13192S
*SLC39A9/Sigma/HPA075390
*C3orf58/Proteintech/27145-1-AP
*C3orf58/Medimabs/home-made
*GAPDH/Novus Biologicals/NB300-320
*LAMP2/Santa Cruz Biotechnology/sc-18822
*Nanog/Abcam/ab21624
*Tra1-60/Stemcell Technologies/60064
*SSEA-4/Santa Cruz Biotechnology/sc-21704
*OCT3/4/Santa Cruz Biotechnology/sc-8628
*MERTK/R&D Systems/MAB8912
*CSF1R/R&D Systems/MAB329
*CX3CR1/Biolegend/341604
*NDST1/Abnova/H00003340-M01A

Validation

*target/company/cat #
*Heparan sulfate (10e4)/AMSbio/370255-1
- CiteAb profile confirms reactivity in human samples and WB and ICC applications (https://www.citeab.com/antibodies/889828-h1890-heparan-sulfate-10e4-epitope?des=e10ef8621e4b3be1)
*HA tag/Biolegend/902301
- CiteAb profile confirms reactivity in human samples and WB and ICC applications (https://www.citeab.com/antibodies/2862337-901501-purified-anti-ha-11-epitope-tag-antibody?des=4b7b1eb6146d111d)
*HA tag/Cell Signaling/2367S
- CiteAb profile confirms reactivity in human samples and WB and ICC applications (https://www.citeab.com/antibodies/123262-2367-ha-tag-6e2-mouse-mab?des=f6f11c593e9fb967)
*GM130/BD Biosciences/610823
- CiteAb profile confirms reactivity in human samples and ICC applications (https://www.citeab.com/antibodies/2412252-610823-bd-transduction-laboratories-purified-mouse?des=a8d5dff81d3685dd)
*Golgin 97/Cell Signaling/13192S
- CiteAb profile confirms reactivity in human samples and ICC applications (https://www.citeab.com/antibodies/2043114-13192-golgin-97-d8p2k-rabbit-mab?des=cb5fb9da100fc262)
*SLC39A9/Sigma/HPA075390
- We confirm reactivity and specificity in human samples by Western blot in Supplementary Figure 7D.
*C3orf58/Proteintech/27145-1-AP
- We suggest low-level reactivity against the overexpressed protein in human RPE-1 cells by Western blot (Supplementary Figure 7C), but did not find that the antibody specifically detects the endogenous protein as the band observed at the expected size is not lost in our KO cell line.
*C3orf58/Medimabs/home-made
- Our Supplementary Figure 21E shows reactivity and specificity by WB against the overexpressed protein in human RPE-1 cells, but no reactivity against the endogenous protein was observed.
*GAPDH/Novus Biologicals/NB300-320
- CiteAb profile confirms reactivity in human samples and WB applications (https://www.citeab.com/antibodies/454922-nb300-320-gapdh-antibody?des=3b2c615a3b9231aa)
*LAMP2/Santa Cruz Biotechnology/sc-18822
- CiteAb profile confirms reactivity in human samples and WB applications (https://www.citeab.com/antibodies/802812-sc-18822-lamp-2-antibody-h4b4?des=75c55049daf633b9)
*Nanog/Abcam/ab21624
- CiteAb profile confirms reactivity in human samples and ICC applications (https://www.citeab.com/antibodies/768634-ab21624-

anti-nanog-antibody?des=a5ebcbe9ca88209e)
*Tra1-60/Stemcell Technologies/60064
- CiteAb profile confirms reactivity in human samples and ICC applications based on information from supplier (https://www.citeab.com/antibodies/3504602-60064-anti-human-tra-1-60-antibody-clone-tra-1-60r?des=1de354a2482cce94)
*SSEA-4/Santa Cruz Biotechnology/sc-21704
- CiteAb profile confirms reactivity in human samples and ICC applications (https://www.citeab.com/antibodies/837387-sc-21704-ssea-4-antibody-813-70?des=fde2bf25f5a503f7)
*OCT3/4/Santa Cruz Biotechnology/sc-8628
- This publication confirms reactivity in human samples and ICC applications (https://pmc.ncbi.nlm.nih.gov/articles/PMC3138706/)
*MERTK/R&D Systems/MAB8912
- CiteAb profile confirms reactivity in human samples and FACS applications (https://www.citeab.com/antibodies/700766-mab8912-human-mer-antibody?des=1213a09e8486dd16)
*CSF1R/R&D Systems/MAB329
- CiteAb profile confirms reactivity in human samples and FACS applications (https://www.citeab.com/antibodies/698803-mab329-human-m-csf-r-cd115-antibody?des=71469fe62c1479e0)
*CX3CR1/Biolegend/341604
- CiteAb profile confirms reactivity in human samples and FACS applications (https://www.citeab.com/antibodies/523484-341604-pe-anti-human-cx3cr1-antibody?des=53bad2f8baeee6a8)
*NDST1/Abnova/H00003340-M01A
- Supplier specifies reactivity against Human, and application in Western blot.

# Eukaryotic cell lines

Policy information about cell lines and Sex and Gender in Research

Cell line source(s)

RPE-1 cells are from ATCC - female
EDi001-A cells are from the European Bank of induced pluripotent Stem Cells (EBiSC) - female

Authentication

none of the cell lines were authenticated

Mycoplasma contamination

Cell lines were negative for mycoplasma contamination

Commonly misidentified lines
(See ICLAC register)

n.a.

# Plants

Seed stocks

*Report on the source of all seed stocks or other plant material used. If applicable, state the seed stock centre and catalogue number. If plant specimens were collected from the field, describe the collection location, date and sampling procedures.*

Novel plant genotypes

*Describe the methods by which all novel plant genotypes were produced. This includes those generated by transgenic approaches, gene editing, chemical/radiation-based mutagenesis and hybridization. For transgenic lines, describe the transformation method, the number of independent lines analyzed and the generation upon which experiments were performed. For gene-edited lines, describe the editor used, the endogenous sequence targeted for editing, the targeting guide RNA sequence (if applicable) and how the editor was applied.*

Authentication

*Describe any authentication procedures for each seed stock used or novel genotype generated. Describe any experiments used to assess the effect of a mutation and, where applicable, how potential secondary effects (e.g. second site T-DNA insertions, mosiacism, off-target gene editing) were examined.*

# Flow Cytometry

## Plots

Confirm that:

☒ The axis labels state the marker and fluorochrome used (e.g. CD4-FITC).

☒ The axis scales are clearly visible. Include numbers along axes only for bottom left plot of group (a 'group' is an analysis of identical markers).

☒ All plots are contour plots with outliers or pseudocolor plots.

☒ A numerical value for number of cells or percentage (with statistics) is provided.

## Methodology

Sample preparation

For RPE-1 cells:
Cells were harvested by trypsinization, pooled by repetitive centrifugation, counted, and split in 5x 15 mL tubes containing 30x10^6 cells each (>150x10^6 cells per replicate). These were spun down and gently resuspended in 4 % PFA/PBS (pH 7.4, 10 mL/30x10^6 cells) by slowly vortexing. After a 15 min incubation on a rotator, cells were spun down and rinsed 3 times in PBS by repetitive resuspension/centrifugation. The cell suspensions were kept in 5 mL PBS at 4°C in the dark before proceeding to triage by FACS. For triage, cell suspensions were adjusted to 30x10^6 per 2 mL and dispensed in 5mL FACS tubes with a cell strainer snap cap (Corning, 352235). The sorted cells for each population (replicate 1 – Low PFF/High PFFs;

| | |
|---|---|
| | replicate 2 – Low PFF/High PFF) were pooled, centrifuged, and the cell pellet was kept at -80°C before gDNA extraction.<br><br>For iPSC-derived microglia:<br>For quality control of differentiation by FACS, cells were blocked with Human TrueStain FcX and TrueStain Monocyte Blocker (Biolegend) and stained with the following antibodies: anti-CSF1R (clone #61708, R&D Systems), anti-CX3CR1 (clone #2A9-1, Biolegend), anti-MERTK (clone #125518, R&D Systems). Dead cells were excluded based on LIVE/DEAD Fixable Aqua (ThermoFisher Scientific) staining. |
| Instrument | RPE-1 cells: BD Biosciences FACS Aria II (replicate 1 of the screen), or BD Biosciences Influx Cell sorter System (replicate 2 of the screen).<br>Microglia: ThermoFisher Scientific Attune Nxt Flow Cytometer |
| Software | FlowJo was used for analysis/visualization of microglia-related data, and for |
| Cell population abundance | For RPE-1-related experiments: In total, 10x10^6 to 13x10^6 cells per sample (replicate 1 – Low PFF/High PFFs; replicate 2 – Low PFF/High PFF) were obtained after sorting, providing 138-fold library coverage. Purity is irrelevant.<br><br>For microglia: N/A. No sorting was performed. |
| Gating strategy | RPE-1 cells - screen replicate 1 & 2: the gating strategy, and boundaries between PFF positive and negative cells is provided in Supplementary Figure 22. Briefly, appropriate forward and side scatter profiles were used to exclude debris and doublets from the analysis (P1, P2, P3), then PFF positive and negative populations were defined based on comparison between PFF-treated and untreated cells. The sorting strategy was set by using gates emcompassing cells having the top 15% or bottom 15% PFF-A633 fluorescence in the screen population.<br><br>Microglia: appropriate forward and side scatter profiles were used to exclude debris and doublets from the analysis. Live cells were analyzed, corresponding to the lower 98.7% fluorescent cells based on LIVE/DEAD Fixable Aqua stain. |

☒ Tick this box to confirm that a figure exemplifying the gating strategy is provided in the Supplementary Information.
