# Supplementary material for: Novel regulators of heparan sulfate proteoglycans modulate cellular uptake of α-synuclein fibrils
Source: Commun Biol. 2025 Oct 6;8:1426. doi: 10.1038/s42003-025-08786-2 (PMC12501064; doi:10.1038/s42003-025-08786-2)
Supplement: Supplementary file 6 — Description of Additional Supplementary Files [file 42003_2025_8786_MOESM6_ESM.pdf]

Description of Additional Supplementary Files

File name: Supplementary Data 1

Description: contains Supplementary Tables 1-9

File name: Supplementary Data 2

Description: All source data underlying the graphs and charts presented in the main and supplementary figures
